# Characterisation of guided entry of tail-anchored proteins in *Magnaporthe oryzae*

**DOI:** 10.1371/journal.ppat.1013011

**Published:** 2025-07-28

**Authors:** Felix Abah, Qiaojia Zheng, Xinru Chen, Linwan Huang, Xiaomin Chen, Jules Biregeya, Osakina Aron, Zonghua Wang, Wei Tang

**Affiliations:** 1 State Key Laboratory of Agricultural and Forestry Biosecurity, College of Plant Protection, Fujian Agriculture and Forestry University, Fuzhou, China; 2 Fujian Universities Key Laboratory for Plant-Microbe Interaction, College of Life Science, Fujian Agriculture and Forestry University, Fuzhou, China; 3 Fujian Provincial Key Laboratory on Conservation and Sustainable Utilization of Marine Biodiversity, Fuzhou Institute of Oceanography, Minjiang University, Fuzhou, China; Purdue University, UNITED STATES OF AMERICA

## Abstract

Rice (*Oryza sativa* L.) is one of the most important staple foods for human population worldwide. However, rice production continues to be severely threatened by rice blast disease caused by an ascomycete fungus *Magnaporthe oryzae*. Tail-anchored (TA) proteins are conserved across diverse organisms and belong to a class of polypeptides that are inserted into the membrane by a hydrophobic sequence located at the C-terminal region. The Guided Entry of Tail-anchored (GET) complex is responsible for the post-translational insertion of nascent TA proteins into the *Saccharomyces cerevisiae* ER lipid bilayer. In *S. cerevisiae,* the GET pathway comprises six known associated components Get1, Get2, Get3, Get4, Get5, Sgt2 and Ssa1 that have been identified and extensively studied. However, the role of the GET complex in rice blast fungus has not been elucidated. Here, we identified five proteins of the GET Complex in *M. oryzae*, namely MoGet1, MoGet2, MoGet3, MoGet4 and MoSgt2 and generated the gene knock-out mutants. Deletion of *MoGET1* and *MoGET2* revealed that they are required for vegetative growth, asexual reproduction, pathogenesis, and right localization of TA protein, MoYsy6, while MoGet3 negatively regulates hyphal growth, asexual development and pathogenesis of *M. oryzae*. In contrast, loss of MoGet4 and MoSgt2 had no effect on the normal development of the rice blast fungus. We demonstrated that the MoGet2 is important in osmotic stress response and positively regulates cell wall integrity. The MoGet1 and MoGet2 were ER-localized and indispensable for DTT-induced ER stress response. *In vitro* and *in vivo* interaction assay revealed MoGet3 has physical interaction with both MoGet1 and MoGet2, indicating the existence of a possible synergistic function amongst the Get components in rice blast fungus. In summary, this finding provides valuable insight into the biological functions of the GET components in plant fungal pathogens.

## Author summary

Rice blast disease, caused by *Magnaporthe oryzae* is a devastating disease of rice, causing major economic loss in rice production across the globe. Since its isolation in 1892, *M. oryzae* has also been implicated in infecting other grasses as wheat, finger millet and barley. *M. oryzae* initiates pathogenicity by forming a specialized, dome-shaped appressorium when in contact with the hydrophobic surface of its host. The Guided Entry of Tail-anchored (GET) pathway, known to direct tail-anchored proteins to their target organelles in yeast and mammals, remains unexplored in pathogenic fungi. Here, we dissected the role of the GET pathway in *M. oryzae* pathogenesis using gene knockout strategies. We showed that while MoGet4 and MoSgt2 of the pathway has no effect on the virulence, ER-localized MoGet1 and MoGet2 proved critical for the pathogenesis of *M. oryzae*. Additionally, we demonstrated that MoGet2 is especially essential for stress tolerance and cell wall integrity, traits vital for host invasion. In contrast, MoGet3 is a negative regulator of vegetative growth and pathogenicity. We also demonstrated through yeast two-hybrid assay and co-immunoprecipitation that MoGet1, MoGet2 and MoGet3 interact with one another *in vitro* and *in vivo*, suggesting a coordinated regulatory network.

## Introduction

Rice (*Oryza sativa* L.) production generates income and employment for more than 200 million households around the world [1], especially for low-income earners in rural and semi-urban areas in Africa, Asia, Latin America and the Caribbean [2]. Total rice consumption is expected to increase to around 584 million tonnes by 2050 [3]. However, rice production is severely affected by rice blast disease, caused by the filamentous fungus *Magnaporthe oryzae* (anamorph: *Pyricularia oryzae*). Rice blast is a widespread disease that affects rice production around the world and therefore poses a threat to global food security [4]. The annual yield loss due to rice blast are around 30% worldwide [4–6], which corresponds to a quantity that can feed 60 million people [7,8]. In addition, *M. oryzae* causes blast disease in other grass species, including barley and wheat (*Triticum aestivum*) [8,9].

To initiate blast disease, the three-celled, teardrop-shaped conidium of *M. oryzae* attaches firmly to the surface of the host (with the help of strong, glycoprotein-rich mucilages) [10] and germinates under favorable conditions [11]. A highly melanized and turgor-pressurized dome-shaped infection cell, the appressorium, then forms at the tip of the germ tube. A penetration peg emerges from the dome-shaped appressorium, which penetrates the plant cuticle and cell wall and branches into invasive hyphae [4,11]. A mature appressorium is highly melanized and chitin-rich, and accumulates glycerol to generate about 8.0 MPa turgor pressure used to mechanically breach the hydrophobic host surface [12].

Tail-anchored (TA) proteins are a class of proteins possessing C-terminal hydrophobic trans-membrane domains [13], and are conserved across diverse organisms, including bacteria, *S. cerevisiae*, *Homo sapiens, Toxoplasma gondii* and plants [14]. They share a topology of cytosolic N-terminal region and a transmembrane domain (TMD) at their C termini [14–16]. However, they lack N-terminal signal peptide, and are therefore targeted to the membrane by posttranslational mechanisms [17]. They are implicated in determining organellar identity and mostly play fundamental roles in cellular metabolism and organismal survival [17–19]. However, malfunction of these proteins may result in disease and aging [20].

Generally, unlike soluble proteins, newly synthesized integral membrane proteins face several challenges, such as aggregation or inappropriate interaction with other proteins in the cytosol, misfolding resulting in non-functional or potentially harmful protein structures, and mislocalization to unintended destinations, leading to protein dysfunction [21]. These phenomena occur as the nascent proteins traverse the aqueous cytosolic environment before reaching their membrane destination [21] and pose acute challenges to protein homeostasis in the living cell [22].

To address these challenges, cells have evolved various mechanisms to ensure the proper biogenesis and trafficking of integral membrane proteins [14]. These mechanisms involve the assistance of molecular chaperones, signal sequences, and protein translocation machinery. This compartmentalization and protein quality control help minimize the risk of misfolding, aggregation, and mislocalization during the biogenesis of integral membrane proteins.

In the past decades, different protein-delivering pathways that deliver proteins to different organelles such as the ER, mitochondria, chloroplasts, and peroxisomes, have been extensively studied and described. The sorting of TA proteins through these different pathways is guided by physicochemical properties such as length and hydrophobicity index of TMDs, and charge of C-terminus, but not by motif sequence [14,17].

The Guided Entry of the Tail-Anchored Protein (GET) Pathway is highly conserved in all eukaryotic organisms [19,23] and functions to post-translationally integrate TA proteins into the membrane of the ER [24,25]. The GET pathway has about 6 (six) known associated component factors for the recognition, shielding, trafficking and insertion of newly synthesized protein substrate into the ER lipid bilayer. In budding yeast, the transmembrane domain (TMD)-recognition complex (TRC) Get4 recruits Get3, Get5 recruits Sgt2, and Sgt2 can recruit other chaperones [19,25–27]. Get3 transfer TAs to Get1/Get2 insertase in the ER lipid bilayer. The pathway was first identified and studied in yeast, followed by mammals, where work on a model animal, *Mus musculus*, identified a 40-KDa ATPase, Transmembrane Recognition Complex (TRC40) [21]. TRC40 is the homolog of Get3 (guided entry of tail-anchored proteins factor 3, ATPase) in yeast [28]. Furthermore, in *Fusarium graminearum*, FgGET3 was reported to be essential for vegetative growth, polar growth, vacuole fusion, conidia production, morphology and germination, stress responses, pathogenicity, and reduced DON production [29].

In rice blast fungus, neither the Ssa1 protein nor the GET/TRC pathway homologs have been theoretically identified or experimentally validated. Therefore, this study first identified the *M. oryzae* homologs of Get1, Get2, Get3, Get4 and Sgt2 based on the sequence, structure and functional similarity to the Gets of *S. cerevisiae* origins. However, we could not identify the *M. oryzae* homolog of the Get component, Get5/UBL4A. Nonetheless, we believe this is the first study that provides the functional characteristics of GET machinery in rice blast fungus, *M. oryzae,* and will establish the broader significance of the GET pathway in pathogen virulence.

## Materials and methods

### Fungal strain, plant and culture condition

The WT Guy11 (obtained from the Fungal Genetics Stock Centre, FGSC 9462) strain was used for the generation of the deletion mutants *(*∆*Moget1*, ∆*Moget2*, ∆*Moget3*, ∆*Moget4* and ∆*Mosgt2*). Plants for infection assays included the susceptible rice cultivar (*O. sativa* CO-39) and barley cultivar, Golden Promise.

The WT Guy11, mutants and the complemented strains were cultured in the complete medium (CM: 6 g yeast extract, 6 g casein hydrolysate and 10 g sucrose in 1 L ddH_2_O) and complete medium II (CM II: 50 mL 20 × Nitrate salt, 1 mL 1000 × trace elements, 1 mL1000 × vitamin solution, 10 g D-glucose, 2 g peptone, 1 g casein hydrolysate, 1 g yeast extract and 15 g agar powder in 1 L ddH_2_O). Other media used include oatmeal agar (OA: 40 g of oatmeal granules and 20 g of agar powder in 1 L of ddH_2_O), rice straw decoction (SDC: 100 g rice straw and 20 g agar powder in 1 L of ddH_2_O), rice bran (RB: 40 g rice bran and 20 g agar powder in 1 L of ddH_2_O), minimal medium (MM: 6 g NaNO_3_, 0.52 g KCl, 0.312 g MgSO_4_.7H_2_O, 1.52 g KH_2_PO_4_, 0.01 g Vitamin B1, 1 mL 1000 × trace elements, 10 g D-glucose and 20 g agar powder in 1 L of ddH_2_O) and terrific broth 3 (TB3: 6 g casein hydrolysate, 6 g yeast extract, 200 g sucrose and 20 g agar powder in 1 L ddH_2_O). All the media used in this experiment were autoclaved for 20 min at 121^o^C. Autoclaved media were allowed to cool to about 50°C and dispensed into sterile 70 × 15-mm Petri plates at about 15 ml per plate. Each dish was inoculated in the center with a block of agar from the stock culture of Guy11 or the mutant strains. Unless otherwise stated, all the cultures were incubated at 26°C under diurnal fluorescent light (12/12-h light/darkness cycle). Medium treatments have three independent biological experiments with five technical replicates each time, unless otherwise stated. Colony diameters were measured after 10 days of incubation. All the pH was maintained at 7.0 unless otherwise stated.

The *Escherichia coli* strain (DH5α) was used to propagate vectors. *DH5α* was grown in liquid or on solid Lysogeny broth (LB: 10 g tryptone, 5 g yeast extract and 10 g NaCl in 1 L of ddH_2_O. pH 7.0) supplemented with or without ampicillin antibiotic (Solarbio Tech. Co., Ltd, Beijing, China).

Plasmids pCX62 (for gene knockout), pKNTG-GFP and pYF11 (for complementation and protein expression) and RFP-HDEL (for expression and colocalization study) used in this study were sourced from the State Key Laboratory for Plant-Microbe Interaction, Plant Protection College, Fujian Agriculture and Forestry University, Fuzhou 350002, China.

### Bioinformatic analysis

For the identification of candidate components of Get proteins in *M. oryzae*, the amino acid sequences of GETs (ScGet1, ScGet2, ScGet3, ScGet4 and ScSgt2) from *Saccharomyces cerevisiae* were used as references to perform Blastp similarity search against *M. oryzae* proteome on NCBI (https://www.ncbi.nlm.nih.gov/) database accessed on 1 December 2022) and validated on FungiDB (https://fungidb.org, accessed on 2 December 2022). The identified amino sequences were subsequently named as MoGet1, MoGet2, MoGet3, MoGet4 and MoSgt2 respectively. The Get proteins of other fungi were identified the same way as the *M. oryzae* Get component proteins. SMART database (http://smart.embl-heidelberg.de) was used for domain prediction and was accessed on 30 November 2023. Finally, GPS IBS [30] and MEGA7 [31] softwares were used to construct the domain architecture and phylogenetic tree, respectively, of the proteins [12,32].

### Generation of *M. oryzae* targeted gene deletion mutants

To generate deletion mutants, homologous recombination approach was adopted to replace the target genes (*MoGET1*, *MoGET2*, *MoGET3*, *MoGET4* and *MoSGT2*) with hygromycin-resistant fragment as previously described [12,33,34]. Briefly, the upstream (A fragment) and downstream (B fragment) flanking regions of the genes were amplified from *M. oryzae* genomic DNA using the primer pairs AF/AR and BF/BR, respectively. Also, HY and YG fragments of hygromycin phosphotransferase (*HPH*) gene were amplified from the pCX62 vector using primer pairs HYG-F/HY-R and YG-F/HYG-R, respectively. The A and B fragments were then fused with HY and YG divisions to obtain AH and BH fragments, respectively, by simultaneous overlap extension PCR (SOE-PCR). All the fragments were amplified using Phanta Max Super-Fidelity DNA polymerase kit (Vazyme Biotech Co. Ltd, Nanjing, China). All primers used in this study are listed in S1 Table.

Protoplast was isolated from wild type Guy11 strain and genetic transformation was conducted using Polyethylene glycol (PEG)-mediated transformation method as previously described [35]. Candidate transformants were selected on TB3 solid media supplemented with 100 µg/mL hygromycin B or G418. Putative transformants were screened by PCR using primer pairs OF/OR and AUF/H853 or OR-F and GFP-R and the positive transformants confirmed by southern blot analysis (S1 Fig).

### Generation of complementation strains

Complementation strains for ∆*Moget1*, ∆*Moget2*, ∆*Moget3*, ∆*Moget4* and ∆*Mosgt2*, were generated by amplifying the entire ORFs sequences of *MoGET1*, *MoGET2*, *MoGET3*, *MoGET4* and *MoSGT2* and their respective native promoter from the WT Guy11 strain. Targeted bands were purified and cloned behind GFP in pYF11 plasmid and constructs were transformed into the protoplasts of their respective mutant strains. The transformants were then screened by PCR and confirmed by Southern blot analyses.

To further determine the subcellular localization of MoGet1 and MoGet2 in *M. oryzae*, we constructed the GFP expression vectors pYF11::MoGet1 and pYF11::MoGet2 of MoGet1 and MoGet2, respectively, and introduced them into the wild-type strain. The transformants were screened using PCR and ascertained by laser confocal microscopy. Further, RFP-HDEL (endoplasmic reticulum Marker protein) was introduced into the respective positive transformants obtained to ascertain the organellar localization of the MoGet1 and MoGet2.

### DNA extraction, gel electrophoresis and southern blot analysis

Fungal genomic DNA was extracted from lyophilized mycelia of the Guy11, mutants and complemented strains using cetyltrimethylammonium bromide (CTAB) [32] protocol. The DNA concentration and purity were measured using Nanodrop (ThermoFisher Scientific, Basingstoke, UK). Gel electrophoresis, enzymatic DNA digestion and purification, ligation and Southern blot hybridization was conducted according to the procedures described previously [32,35]. Probing, hybridization, staining and balance were performed using the DIG HIGH Prime DNA Labelling and Detection Starter Kit I (Roche Diagnostics GmbH, Mannheim, Germany).

### Vegetative growth, osmotic stress, cell wall integrity and reactive oxygen species sensitivity analysis

For vegetative growth assay, the WT Guy11, the GETs-deficient mutants and their corresponding complemented strains were cultured on CM, CM II, OA, SDC and MM solid media. The cultures were incubated at 26^o^C, 45% relative humidity (RH) in diurnal light for ten days. The diameter of each colony was measured using a meter rule in two perpendicular dimensions and the average of the two measurements was taken after subtracting the 5 mm diameter of the colonized plug.

For sensitivity assays, wild-type Guy11 strain and the mutant strains were inoculated on CM II solid media supplemented separately with osmotic stressors (NaCl, KCl and Sorbitol), cell wall and membrane stressors (CR, CFW, SDS and DTT) and 5 mM ROS H_2_O_2_ agent; and incubated for 10 days at 26^o^C, 45% RH and 12 h light/12 h dark photoperiod. Colony diameters was evaluated between treated and non-treated groups.

To examine the cell wall integrity, the WT and the mutant strains ∆*Moget1*, ∆*Moget2* and ∆*Moget3* were treated with lysis enzyme following the previously established protocol [32,35,36] with slight modification. Briefly, 3-day old mycelia grown in CM liquid media was ground using a sterile laboratory mortar and transferred into fresh CM media. The culture was re-incubated at 26^o^C, 110 rpm for 12 hrs. Mycelia were filtered and 0.2 g lysing enzyme (SIGMA-ALDRICH Co., St. Louis, USA) in 20 mL 1 M C_6_H_14_O_6_ (sorbitol) added to 2 g of the wet weight mycelia. Protoplast release by each strain was estimated using haemocytomer. All the experiments were conducted in a sterile laminar flow hood. All the experiments were repeated 3 times, with 3 replicates each time.

### Conidiophore formation, conidiation and pathogenicity assay

Conidiophore development assays were performed by inoculating 5-day old mycelial plugs of Guy11, the mutants under study and their complemented strains on conidia-inducing rice bran agar media and incubated them at 26^o^C for 7 days. The aerial mycelium of each strain was scrubbed off and about 1 cm × 0.5 cm mycelial plug was excised and laid on a slide. The slide was placed in 90-mm Petri plate, incubated at 27^o^C under humid conditions, and observed under light microscope at different time points of 12 -, 24 - and 48 hrs. For conidiation assay, the cultures were re-intubated in continuous white fluorescent light for 3 days at 27^o^C after scrapping off of the vegetative mycelia mass to induce sporulation. Spore suspension was prepared from each strain and estimated independently under light microscope using haemocytometer.

To test the pathogenicity of each strain, an edge of growing 5-day old mycelia of each strain was excised and inoculated on 10-day old barley leaf (Gold Promise cultivar) and incubated in the dark for 24 hrs and then transferred to fluorescence continuous light at 27^o^C for 6 days. Similarly, spore suspensions prepared from wild type control, ∆*Moget3*, ∆*Moget4* and ∆*Mosgt2* were spray or punch inoculated on 3-week or 6-week old rice leaves. The infected seedlings were incubated in the dark for 24 hrs and diurnal for 5 days at room temperature and about 85% RH.

### *In vivo* penetration assay and life cell imaging

Host penetration and invasive hyphal growth assays were examined by inoculating 10-day old barley leaves and incubated in the dark at 26^o^C and under humid conditions. The leaf sheet was peeled at different time point of 12 -, 16 - and 24 hpi and examined under microscope as described previously [12,37].

### RNA extraction for qPCR

To study the expression of hydrophobin genes in ∆*Moget1/*∆*Moget2*, 3-day-old mycelia samples of Guy11 and ∆*Moget1/*∆*Moget2* were grown in CM broth at 26^o^C and 110 rpm. Mycelia were then filtered and washed twice using double distilled water. Total RNA was extracted from the samples using the Eastep Super RNA extraction kit (Promega Biotech Co. Ltd, Beijing, China) according to the manufacturer’s instructions. cDNA was synthesized from the total RNA by reverse transcription PCR using the Evo M-MLV RT kit with gDNA clean for qPCR (Accurate Biotechnology Co. Ltd, Hunan, China) according to the manufacturer’s instructions. Fluorescence quantitative real-time PCR was conducted using ChamQ Universal SYBR qPCR Master Mix as recommended by the manufacturer (Vazyme Biotech Co. Ltd, Nanjing, China). Three biological replicates with 3 technical replicates per biological replicate was applied to the experiments.

### Yeast two-hybrid assay

Yeast two-hybrid analysis to examine the interaction of MoGet1, MoGet2 and MoGet3 was performed using the MATCHMAKER GAL4 two-hybrid system 3 (Takara Bio, San Jose, USA). The protein-coding regions of the three genes were amplified from Guy11 wild-type cDNA with the primer pairs (S1 Table). MoGet1, MoGet2 and MoGet3 were cloned in the pGBKT7 bait vector, while MoGet1 or MoGet3 in the pGADT7 as prey vector. The pGBKT7 and pGADT7 were digested with *Nde* I and *EcoR* I restriction enzymes, as described previously [38]). The AD and BD constructs were cotransformed into AH109 *S. cerevisiae* strain [39]. pGBKT7–53/pGADT7-T and pGBKT7-Lam/pGADT7-T vectors were used as positive and negative controls, respectively. The emerged yeast colonies in SD/-Leu/-Trp media were isolated and cultivated on SD-four-deficient selective media (SD/-His-Leu-Trp-Ade) supplemented with 40 µg/mL X-α-gal for color development.

### Protein extraction, co-immunoprecipitation (Co-IP) and western blot assay

Total protein was extracted from 3-day old *M. oryzae* mycelia of MoGet1-RFP, MoGet3-RFP, MoGet1-GFP and GFP-MoGet2 according to previous protocol [40]. Briefly, about 5 g mycelial powder prepared by grinding lyophilized 3-day old mycelia was added into a sterile 2-mL Eppendorf tube. 1 mL Lysis buffer (10 mM Tris/Cl pH = 7.5, 150 mM NaCl, 0.5 mM EDTA, 0.5% NP-40), 10 μL PMSF, 10 μL protein inhibitor in the ratio of 100:1:1 were added. The suspension was vortexed to homogenize, incubated in ice for 10 min and centrifuged at 4^o^C, 14000 rpm for 15 min (Micro-centrifuge 5430 R). The supernate was collected into a 1.5-ml Eppendorf centrifuge tube and 5x SDS buffer was added at the ratio of 5 mL supernate to 1 μl 5x SDS buffer. The sample was boiled for 10 min for denaturing and stored at -20^o^C for western blot analysis.

For Co-IP assay, GFP-fusion proteins were isolated and incubated with 30 μl GFP-Trap magnetic beads (ChromoTek, Martinsried, Germany) according to manufacturer’s instructions. To obtain the protein from GFP-Trap magnetic beads, the sample-containing 10-mL tubes were placed in ice for 10 min for magnetic beads sedimentation. Then, the tubes were transferred to a magnetic rack, allowed for 1 min to trap the GFP-bound beads and the supernate was carefully discarded. The beads were washed three times with dilution buffer (50 mM Tris, 150 mM NaCl, pH 7.4) and then elusion buffer. Equal volume of protein loading buffer was added, then denatured by boiling and placed on a magnetic frame for 1 min. The elusion protein was collected and 40 μl of total protein was loaded in 10% SDS-PAGE gel for immunoblotting and co-immunoprecipitation analysis.

For phosphorylation signal, total protein from the mycelial of Guy11, ∆*Moget1*, ∆*Moget2* and ∆*Moget1/*∆*Moget2* double mutant was extracted as previously described [41]. The phosphorylation level of Mps1 was detected through western blot analysis. Briefly, 40 µl of total protein sample was loaded into each 10% SDS-PAGE gel well and separated by electrophoresis. The gel was transferred onto a nitrocellulose membrane (Amersham, Piscataway, NJ, USA) for blotting. The membrane was incubated in p44/42 MAPK antibody (Cell Signaling Technology, Beverly, MA, USA). Chemiluminescent signals of the specific protein bands were detected using ECL kit (Amersham Biosciences, Freiburg, Germany).

### Determination of the fate of TA protein representative, MoYsy6, in GET-deficient mutant

To determine the fate of TA proteins, representative TA protein, MoYsy6 (MGG_04002), was tagged at the C-terminal with GFP using pKNT-GFP as a vector. The construct was transformed into the protoplast of Guy11, ∆*Moget1* and ∆*Moget2* according to the previous protocol [35]. Transformants were selected on TB3 medium supplemented with G418, screened by PCR and confirmed via laser confocal microscopy.

### Microscopic observation

Hyphal penetration and invasion on host media surfaces, GFP and RFP localizations were observed under a laser confocal microscope equipped with a Nikon A1 Plus imaging instrument (Tokyo, Japan**).**

### Statistical analysis and reproducibility

Quantification of growth diameter, sensitivity of the strains to stressors, conidiation and lesion types were performed using Microsoft excel spreadsheet and GraphPad prism version 5.01 (GraphPad Software Inc., La Jolla, CA, USA). ImageJ software, Microsoft excel spreadsheet and GraphPad prism5 were used to conduct disease area quantification. Error bars represent standard deviation from the mean in all the figures, and *p* values were determined by one- or two-way ANOVA. All experiments were repeated as stated in each figure legends.

## Results

### Identification of Get proteins in *M. oryzae*

**Fig 1:**
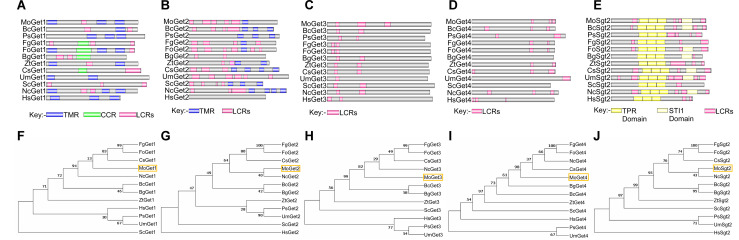
Domain architecture and phylogeny of Gets in phytopathogenic fungi, *Neurospora crassa* and *Homo sapiens.* **(A and F)** Get1 domain architecture and phylogenetic analysis in different fungi. **(B and G)** Get2 domain architecture and phylogenetic analysis in different fungi. **(C and H)** Get3 domain architecture and phylogenetic analysis in different fungi. (D and **I)** Get4 domain architecture and phylogenetic analysis in different fungi. **(E and J)** Sgt2 domain architecture and phylogenetic analysis in different fungi. The evolutionary history was inferred using the maximum likelihood method based on the JTT matrix-based model. The analysis involved 60 amino acid sequences. The maximum likelihood phylogeny for the amino acids was tested with 1000 bootstrap replicates. Evolutionary analyses were conducted using MEGA7. TMR: Transmembrane region; CCR: Coil-coil region; TPR: Tetratricopeptide repeat; STI1: Stress inducible 1; LCRs: low complexity regions.

Amino acid sequences of Get1, Get2, Get3, Get4 and Sgt2 from *Saccharomyces cerevisiae* (S288C) were used to perform a BLASTp search at the FungiDB (https://fungidb.org, accessed on 30 October 2022) and National Centre for Biotechnology Information (https://www.ncbi.nlm.nih.gov/, accessed on 30 October 2022). The identified homologs were named MoGet1, MoGet2, MoGet3, MoGet4 and MoSgt2, respectively. Compare to *S. cerevisiae* Get components, alignment results showed no significant similarity for MoGet1 and MoGet2 (Fig 1A and 1B), but 51.30%, 33.55% and 34.01% sequence similarities were obtained for MoGet3, MoGet4 and MoSgt2, respectively. More homologs of these proteins were found in the other eight top important pathogenic fungi (S2 Table) [42], and further analyzed at SMART database (http://smart.embl-heidelberg.de/, accessed on 14 October 2023). Protein domain prediction showed that MoGet1 and MoGet2 contain transmembrane domain (TMD), unlike cytosolic MoGet3 and MoGet4 that contain only low complexity regions (LCRs) [43,44]. Except for Get1 of *Fusarium graminaerum*, *Blumeria graminis*, *Colletorichum siamense* and *S. cerevisiae* and Get2 of *Blumeria graminis, Ustilago maydis* and *Homo sapiens*, TMD insertase is conserved in all the fungi and mammal analyzed (Fig 1A and 1B). On the other hand, MoSgt2 possesses a TPR catalytic domain, which is conserved in the top nine important plant pathogenic fungi as well as in *N. crassa*, *S. cerevisiae* and *H. sapiens* (Fig 1E). Moreover, the phylogenetic analysis indicates that MoGet1, MoGet2, MoGet3, MoGet4 and MoSgt2 have close ancestry with Gets and Sgt2 in all the fungi analyzed, except that they have distant ancestry with ScGet1, HsGet2, HsGet3, HsGet4 and HsSgt2, respectively (Fig 1F–1J).

To determine the functions of the GETs in *M. oryzae*, we generated their respective knockout mutants by replacing the entire *MoGETs* genes from Guy11 wild type with a hygromycin resistance gene using a homologous recombination approach. Double disruption of **Mo*GET*1** and **Mo*GET2* was performed by transforming the **Mo*GET2* deletion cassette into the ∆*Moget1* background with the bleomycin resistance cassette as the selectable marker. The knockout mutants of the ∆*Moget1*, ∆*Moget2*, ∆*Moget1/*∆*Moget2*, ∆*Moget3,* ∆*Moget4* and ∆*Mosgt2* were then confirmed by a PCR assay and Southern blot analysis (S1 Fig).

### MoGet1 and MoGet2 are crucial for vegetative growth and conidiation

**Fig 2:**
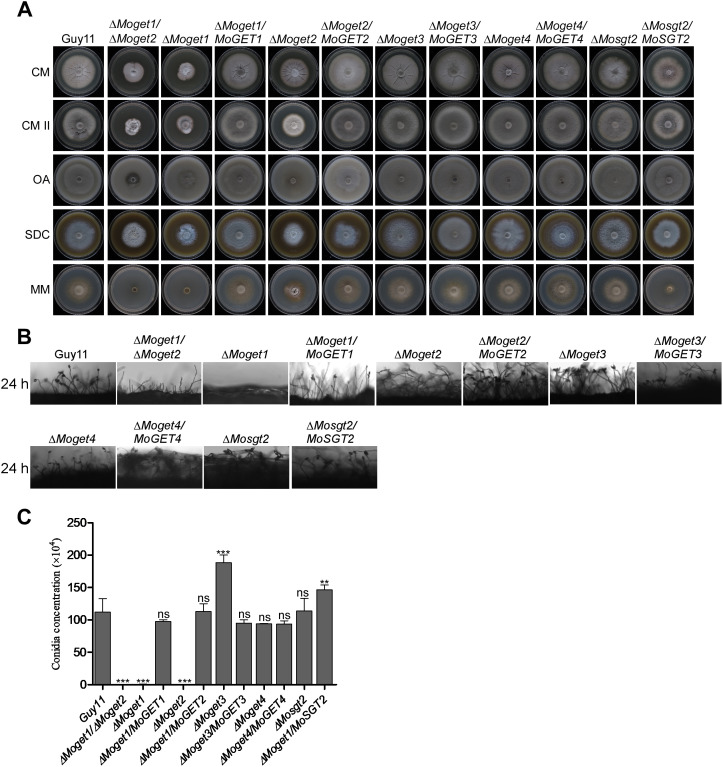
Role of MoGet1, MoGet2, MoGet3, MoGet4 and MoSgt2 in hyphal growth in *M. oryzae.* **(A)** Vegetative growth of the Guy11, mutants and their complemented strains on CM, CM II, OA, SDC and MM media at 10 days post inoculation (dpi). **(B)** Conidiophores and conidia formation of the conidiophores of Guy11, the mutants and complemented strains on rice bran medium. **(C)** Statistical analysis of average conidiation of the respective mutants and their complemented strains relative to Guy11 wild-type strain 10 dpi on RB medium at 27^o^C.

To determine the role of MoGet1, MoGet2, MoGet3, MoGet4 and MoSgt2 in vegetative growth of *M. oryzae*, the ∆*Moget1*, ∆*Moget2*, ∆*Moget1/*∆*Moget2, *∆*Moget3*, ∆*Moget4* and ∆*Mosgt2* strains alongside with the WT Guy11 and the complementation were cultured on CM, CM II, OA, SDC and MM solid media at 26^o^C and colony diameters measured after 10 days. Our results showed that the growths of ∆*Moget1* and ∆*Moget2* mutants were significantly reduced on all the growth media used compared to Guy11 and their respective complemented strains (Fig 2A and Table 1). Conversely, ∆*Moget3* mutant showed a significant increase in vegetative growth compared to the Guy11 wild-type and complemented strains (Fig 2A and Table 1), demonstrating that MoGet3 negatively regulates the fungal vegetative growth. On the other hand, the *MoGET4* and *MoSGT2* gene deletion mutants are similar to Guy11 in vegetative growth. Reintroduction of the *MoGET1, MoGET2*, *MoGET3*, *MoGET4* and *MoSGT2* genes into the mutant strains restored their normal growth, except for the ∆*Moget3/MoGET3* complemented strain which outperformed Guy11 in hyphal growth (Fig 2A). These results demonstrate that MoGet1 and MoGet2 are important for normal vegetative growth, while MoGet3 negatively regulates mycelial development in *M. oryzae*.

**Table 1:** Comparison between the growths of Guy11 and the mutant strains.

	Colony diameter (cm)^g^				
Strain	CM	CM II	OA	SDC	MM
Guy11	4.9 ± 0.33^b^	5.4 ± 0.18^b^	5.1 ± 0.23^b^	5.3 ± 0.16^b^	4.6 ± 0.65^a^
∆*Moget1/* ∆*Moget2*	3.3 ± 0.49^d^	3.3 ± 0.64^d^	3.1 ± 0.33^d^	3.1 ± 0.56^e^	2.8 ± 0.83^c^
∆*Moget1*	2.1 ± 0.63^d^	2.3 ± 0.21^e^	2.7 ± 0.59^e^	2.2 ± 0.79^f^	2.0 ± 0.52^d^
∆*Moget1/* *MoGET1*	5.0 ± 0.22^b^	4.6 ± 1.11^c^	5.1 ± 0.17^b^	4.9 ± 0.52^b^	4.9 ± 0.40^a^
∆*Moget2*	3.9 ± 0.38^c^	3.9 ± 0.49^c^	4.2 ± 0.31^c^	3.9 ± 0.22^d^	3.5 ± 0.31^b^
∆*Moget2/* *MoGET2*	4.9 ± 0.74^b^	4.6 ± 0.40^c^	4.8 ± 0.32^b^	5.0 ± 0.69^b^	4.7 ± 0.50^a^
∆*Moget3*	5.6 ± 0.24^a^	5.8 ± 0.13^a^	5.9 ± 0.095^a^	5.9 ± 0.11^a^	5.2 ± 0.31^a^
∆*Moget3/* *MoGET3*	5.7 ± 0.37^a^	6.1 ± 0.11^a^	6.0 ± 0.04^a^	6.0 ± 0.18^a^	5.2 ± 0.44^a^
∆*Moget4*	5.3 ± 0.45^b^	5.3 ± 0.48^b^	5.8 ± 0.13^a^	5.9 ± 0.11^a^	4.8 ± 0.58^a^
∆*Moget*4*/* *MoGET4*	5.3 ± 0.48^b^	5.4 ± 0.30^b^	5.8 ± 0.15^a^	5.8 ± 0.11^a^	5.0 ± 0.53^a^
∆*Mosgt2*	5.1 ± 0.26^b^	5.2 ± 0.24^b^	5.7 ± 0.083^a^	5.6 ± 0.26^a^	4.8 ± 0.3^a^
∆*Mosgt2/* *MoSGT2*	4.5 ± 0.52^c^	4.7 ± 0.40^c^	5.1 ± 0.33^b^	4.9 ± 0.40^b^	3.2 ± 1.12^b^

^g^Colony diameter (cm) of Guy11, the mutants and their complemented strains on CM, CM II, OA, SDC and MM at 27^o^C 10 dpi.

Mean and standard deviation were determined using contingency table analysis with row means in Microsoft Excel spreadsheets and GraphPad Prism 5. Similar values were obtained from three independent experimental repeats with five technical replicates for each repetition.

The superscript a, b, c, d and e indicate significant changes compare to Guy11. Same letters in a column shows no significant difference.

Conidia facilitate the survival, efficient dissemination and disease perpetuation of the fungus [45]. To unveil the role of the Get proteins in asexual sporulation of *M*. *oryzae*, we harvested, quantified and compared the amount of conidia produced by the various strains. The ∆*Moget1*, ∆*Moget2* and ∆*Moget1/*∆*Moget2* mutant strains failed to produce spores while ∆*Moget4* and ∆*Mosgt2* strains produced similar number of spores as the wild-type strain (Fig 2B and 2C), suggesting that MoGet1 and MoGet2 are essential for conidiation in *M. oryzae*. Conidiophore formation is a prerequisite for conidiation and conidia-mediated infection of host plants under favourable conditions [45]. ∆*Moget1*, ∆*Moget2* and ∆*Moget1/*∆*Moget2* mutants are defective in conidiophore production at 24 hpi (Fig 2B), which further supports their critical involvement in asexual reproduction in *M. oryzae*. Taken together, we conclude that the *MoGET1* and *MoGET2* genes are important for growth, development and asexual reproduction in *M. oryzae*.

### The role of the Get components in stress tolerance

**Fig 3:**
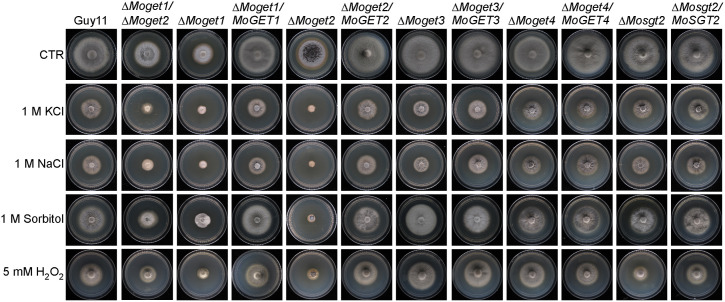
Growth phenotype and sensitivity of MoGETs-deficient mutants to KCl, NaCl, Sorbitol and H_2_O_2_. Guy11 wild-type, ∆*Moget1/*∆*Moget2*, ∆*Moget1*, ∆*Moget2*, ∆*Moget3*, ∆*Moget4*, ∆*Mosgt2* and their complemented strains were cultured on CM II supplemented with 1 M Sorbitol, KCl, NaCl and 5.0 mM H_2_O_2_, incubated at 26^o^C for 10 days and then sampled for sensitivity assay for osmotic and ROS stress response. The inhibition rate of each treatment was compared with the growth rate of the untreated control.

Environmental stimuli trigger adaptive cellular responses to optimize tolerance, survival and proliferation [46,47], and an organism’s response to stress involves the integrated function of many components of cell metabolism [47]. To determine the role of the GET components in stress response in rice blast fungus, we cultured the Guy11, ∆*Moget1*, ∆*Moget2*, ∆*Moget1/*∆*Moget2*, ∆*Moget3*, ∆*Moget4* and ∆*Mosgt2*, and their respective complemented strains on CM II supplemented with 1 M NaCl, KCl and Sorbitol (as osmotic stress-inducing agents) to evaluate their growth responses. It is obvious from the results that the growth of ∆*Moget2* mutant strain was more inhibited on media supplemented with NaCl, KCl and Sorbitol than ∆*Moget1*, ∆*Moget3*, ∆*Moget4* and ∆*Mosgt2* strains (Fig 3 and Table 2). Interestingly, the ∆*Moget1/*∆*Moget2* double knock out mutant was less inhibited than ∆*Moget2*, suggesting that MoGet1 serves as a negative osmo-regulator in *MoGET2*-deficient mutant strain. In sorbitol-supplemented media, ∆*Moget1* was the least inhibited (P < 0.001) strain while ∆*Moget1*/∆*Moget2*, ∆*Moget3* and all the complemented strains showed no significant difference in their growths compared to Guy11. Also, ∆*Moget4,* ∆*Mosgt2* and their complemented strains showed no significant inhibition rate as compared to Guy11 (Fig 3 and Table 2).

**Table 2:** Salt and ROS stress assays of MoGets component mutants.

	Inhibition rate (%)			
	Salt stress^s^			H_2_O_2_ ROS Stress^r^
Strain	KCl	NaCl	Sorbitol	5.0 mM
Guy11	45.16 ± 2.83^c^	43.53 ± 5.32^d^	19.46 ± 5.23^c^	32.64 ± 4.71^c^
*∆Moget1/∆Moget2*	55.50 ± 4.31^b^	57.37 ± 1.66^b^	18.23 ± 5.02^c^	78.96 ± 36.46^a^
*∆Moget1*	47.55 ± 6.01^c^	50.50 ± 8.00^c^	2.90 ± 6.91^d^	25.92 ± 10.69^d^
*∆Moget1/MoGET1*	42.94 ± 4.37^d^	46.63 ± 6.92^c^	15.03 ± 2.09^c^	28.59 ± 10.41^c^
*∆Moget2*	77.86 ± 1.85^a^	80.39 ± 2.26^a^	65.88 ± 13.96^a^	37.29 ± 23.29^c^
*∆Moget2/MoGET2*	45.19 ± 4.93^c^	46.46 ± 5.65^c^	19.26 ± 0.85^c^	26.46 ± 3.92^d^
*∆Moget3*	53.58 ± 0.44^b^	58.96 ± 0.35^b^	16.66 ± 4.21^c^	24.90 ± 5.52^d^
*∆Moget3/MoGET3*	45.73 ± 3.77^c^	49.69 ± 2.30^c^	20.29 ± 0.20^c^	26.06 ± 4.10^d^
*∆Moget4*	40.83 ± 4.05^d^	45.39 ± 3.39^c^	16.34 ± 2.00^c^	24.33 ± 7.66^d^
*∆Moget*4*/MoGET4*	42.45 ± 1.69^d^	40.69 ± 1.50^d^	14.75 ± 1.70^c^	23.56 ± 1.55^d^
*∆Mosgt2*	44.86 ± 2.01^c^	47.92 ± 1.69^c^	20.22 ± 4.08^c^	25.65 ± 6.02^d^
*∆Mosgt2/MoSGT2*	41.94 ± 1.70^d^	46.17 ± 1.70^c^	20.11 ± 3.37^c^	20.86 ± 8.56^d^

s Guy11, ∆*Moget1/*∆*Moget2,* ∆*Moget1*, ∆*Moget2*, ∆*Moget3*, ∆*Moget4* and ∆*Mosgt2* were cultured on CM II supplemented with 1 M Sorbitol, KCl and NaCl and incubated at 26^o^C for 10 days for osmotic stress response.

r Percentage inhibition of vegetative growth of the mutants and complemented strains relative to the Guy11 on CM II supplemented with 5 mM H_2_O_2_ ROS stressor.

Inhibition rate = (the colony diameter of untreated strain minus the colony diameter of the treated strain)/(the colony diameter of untreated strain) * 100. ∆*Moget1*/∆*Moget2* double mutant is highly inhibited in response to ROS unlike the two-single mutants, demonstrating epistatic interactions in which the function of one gene is completely dependent on the presence of a second one.

Mean and standard deviation were calculated using contingency table analysis with row means in GraphPad Prism 5. Similar values were obtained from three independent experimental repeats with three technical replicates for each repetition.

The superscript a, b, c, d and e indicate significant changes compare to Guy11. Same letters in a column shows no significant difference.

Reactive oxygen species (ROS) sensing and metabolism are controlled by a variety of proteins involved in oxidation-reduction reactions [48]. Therefore, we investigated the role of *M. oryzae* Get proteins in oxidative stress response by growing the various strains on CM II medium supplemented with 5 mM H_2_O_2_ at 26^o^C for 10 days [49]. The results revealed that the ∆*Moget1/*∆*Moget2* double mutant was significantly (*** p < 0.001) inhibited on media containing 5 mM H_2_O_2_ compared to the single mutants, their complemented strains and Guy11 (Fig 3, Table 2), suggesting a functional redundancy of the two proteins during oxidative stress response.

**Fig 4:**
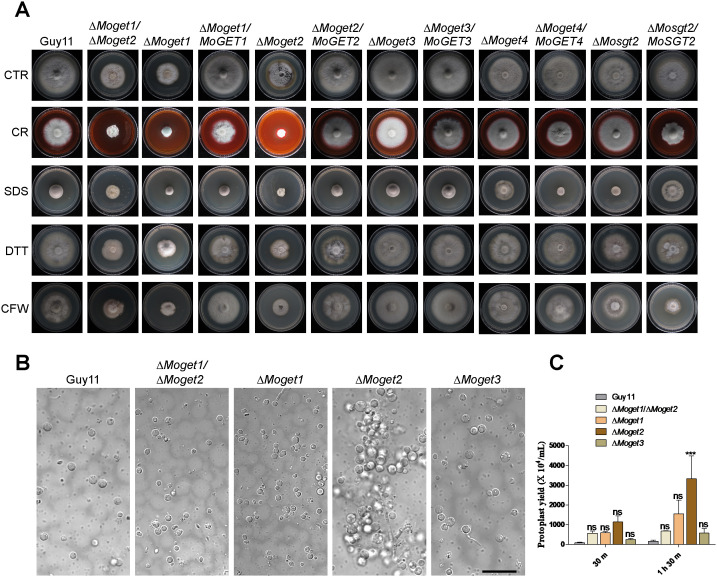
Sensitivity of Guy11, ∆*Moget1/*∆*Moget2*, ∆*Moget1*, ∆*Moget2*, ∆*Moget3*, ∆*Moget4* and ∆*Mosgt2* to cell wall stressors. **(A)** Vegetative growth of the Guy11, the mutants and complemented strains cultured on CM II supplemented with the cell wall stressors (200 µg/mL CR, 0.01% SDS, 2 mM DTT and 200 µg/mL CFW) and imaged 10 dpi. (B) Protoplasts from Guy11, ∆*Moget1/*∆*Moget2,* ∆*Moget1*, ∆*Moget2* and ∆*Moget3* after treatment with lysing enzyme at 30^o^C, 85rpm at different time points: 30 m and 1 h 30 m. Scale bar = 10 µm. **(C)** Amount of protoplast released by the Guy11, ∆*Moget1/*∆*Moget2* double mutant and the three single mutant strains. Statistical analysis was conducted using two-way ANOVA with Bonferroni posttests (GraphPad Prism 5). Each sample was compared with Guy11 wild-type. ‘ns’ and ‘***’ represent significant differences p > 0.05 and p < 0.001, respectively. Error bar represents standard deviation from the mean. The experiments were conducted three times with five independent replicates. ns = no significant difference.

Cell wall maintains cell morphology, protects the cell contents and mediates the transmission of external stimuli into the cell [50]. To establish the contribution of the *GET* genes to cell wall and cell membrane integrity in *M. oryzae*, we cultured the various fungal strains ∆*Moget1*, ∆*Moget2*, ∆*Moget1*/∆*Moget2,* ∆*Moget3*, ∆*Moget4* and ∆*Mosgt2* on CM II media supplemented with the cell wall and cell membrane stressors calcofluor white (CFW), congo red (CR), sodium dodecyl sulphate (SDS) and dithiothreitol (DTT). After 10 days of incubation at 26^o^C, we analyzed the colony diameter of each strain. Results showed that the growth of ∆*Moget3* is less inhibited by CR and CFW than Guy11 (Fig 4A and S3 Table). The ∆*Moget2* mutant, on the other hand, is highly inhibited by CFW, an indication that MoGet2 is required for cell wall integrity in *M. oryzae*. Meanwhile, ∆*Moget1*/∆*Moget2* double mutant was less inhibited by CFW compared to ∆*Moget2* after a 10-day incubation. This result indicates that MoGet1 is necessary for the ∆*Moget2*-induced cell wall compromise. On DTT-supplemented CM II media, our data showed that ∆*Moget1* and ∆*Moget2* are significantly inhibited compared to Guy11. This demonstrates that ER is compromised and MoGet1 and MoGet2 are required for ER membrane integrity.

To further confirm the role of MoGet2 in cell wall maintenance, we exposed the mycelia of Guy11, ∆*Moget1*/∆*Moget2*, ∆*Moget1*, ∆*Moget*2 and ∆*Moget*3 strains to a cell wall-degrading enzyme (25 mg/mL lysing enzyme), cultured under agitation at 85 rpm, 30^o^C for 30 min or 1 h 30 min periods. We observed that the hyphae from the ∆*Moget2* mutant were well digested and released the highest number of protoplasts at each time point, followed by ∆*Moget1* (at 1 h 30 min), ∆*Moget1*/∆*Moget2,* ∆*Moget3* and then Guy11 wild type control in that order (Fig 4B and 4C), indicating that the cell wall of the ∆*Moget2* mutant was more prone to degradation than those of the other strains.

### MoGet1 and MoGet2 are critical for *M. oryzae* pathogenesis

**Fig 5:**
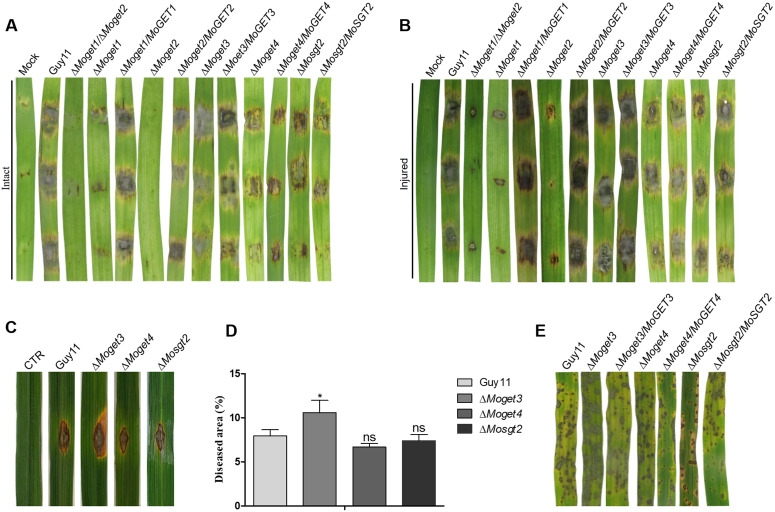
MoGet1 and MoGet2 are critical for virulence of *M. oryzae,* Δ*Moget3* more pathogenic than Guy11. **(A and B)** Disease lesions of Guy11, ∆*Moget1/*∆*Moget2*, ∆*Moget1*, ∆*Moget2*, ∆*Moget3*, ∆*Moget4*, ∆*Mosgt2* and their complemented strains on intact and injured barley leaves, respectively. 10-day old detached barley leaves were wounded (or left intact) with a pipette tip, inoculated with 5-mm mycelial plugs, incubated in the dark for 24 h and then transferred to fluorescence continuous light for 6 days at 27^o^C. **(C)** Disease lesions of Guy11 and spore-producing mutants of ∆*Moget3*, ∆*Moget4* and ∆*Mosgt2* on host rice leaves. 6-week old rice leaves were punch-inoculated, kept in the dark for 24 h and transferred to 12 h/12 h diurnal light under humid conditions. Photograph was taken at 14 dpi. **(D)** Statistical analysis of percentage disease area of representative leaves of Guy11, ∆*Moget3*, ∆*Moget4* and ∆*Mosgt2*. ImageJ software and one-way ANOVA with Tukey’s multiple-comparison test (GraphPad Prism 5) were used to perform statistical analysis. **(E)** Spray inoculation on 3-weeks old host rice leaves using spore suspension prepared from Guy11, ∆*Moget3*, ∆*Moget4* and ∆*Mosgt2.* The inoculated 3-week old rice seedling were maintained in the humid and dark condition for 24 hours and transferred to 12 h/12 h diurnal light. Photograph was taken at 5 dpi. Error bar represents the standard deviation from the mean of three independent repeats and asterisk represents significant differences (P < 0.05).

To investigate the roles of the *GET* genes in the pathogenicity of *M. oryzae*, we inoculated 5-day old mycelial plug from ∆*Moget1*, ∆*Moget2*, ∆*Moget1*/∆*Moget2*, ∆*Moget3*, ∆*Moget4* and ∆*Mosgt2* mutants on 10-day old barley leaves. The infected leaves were observed and photographed 5 days after infection. The results indicate that ∆*Moget1*, ∆*Moget2* and ∆*Moget1/*∆*Moget2* mutants failed to cause noticeable disease symptoms on the susceptible barley leaves (Fig 5A and 5B). However, ∆*Moget3* was significantly more pathogenic than Guy11 and the other strains, suggesting that MoGet3 negatively regulates the pathogenicity of *M. oryzae*.

For conidia-mediated infection, we punch-inoculated 6-week old or spray-inoculated 22-day old seedlings of the susceptible rice cultivar CO-39 with spore suspensions from Guy11 and the various conidia-producing mutants (∆*Moget1*, ∆*Moget2* and ∆*Moget1/*∆*Moget2* do not produce conidia) and assessed the disease symptoms after 10 d for punch inoculation and 5 d for spray inoculation. For punch inoculation, all the strains were able to cause infection, with ∆*Moget3* displaying more expanded disease area (Fig 5C and 5D). Similarly, results of the spray inoculation showed that both ∆*Moget4* and ∆*Mosgt2* exhibited similar virulence, while ∆*Moget3* was more virulent compare to Guy11 (Fig 5E).

### MoGet1 and MoGet2 are crucial for appressorium formation in *M. oryzae*

**Fig 6:**
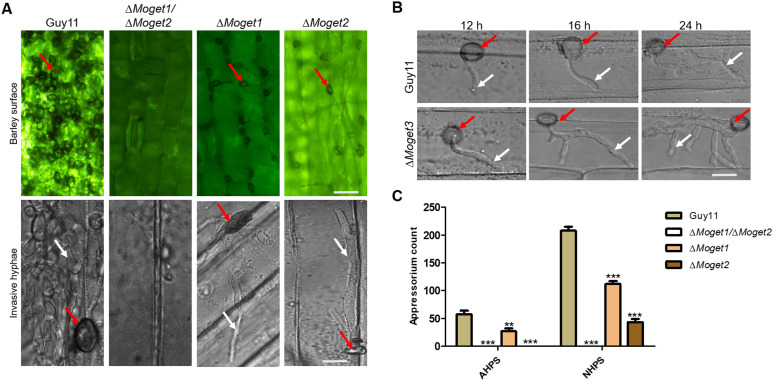
Appressorium formation and penetration assay of Guy11 and the mutant strains. **(A)** Hyphal-mediated appressorium formation of Guy11 wild-type, ∆*Moget1/*∆*Moget2*, ∆*Moget1* and ∆*Moget2* mutant strains on hydrophobic barley surface and their invasion of barley epidermal cell. **(B)** Appressorium-mediated penetration of Guy11 and ∆*Moget3* mutant strains into epidermal cell of barley leaves at 12, 16 and 24 hpi. **(C)** Quantification of hyphal-mediated appressorium formation on artificial and natural hydrophobic surfaces. Error bar represents the standard deviation (SD) from the mean of two independent repeats with two technical replicates. Statistical analysis was conducted using Two-way ANOVA with Bonferroni post-test (GraphPad Prism 5). Each sample was compared with Guy11 wild-type. ‘**’ and ‘***’ represent significant differences p < 0.01 and p < 0.001, respectively. AHPS: Artificial hydrophobic surface; NHPS: Natural hydrophobic surface; Red arrow: Appressorium; white arrow: Hyphae. Scale bar = 10 µm.

Appressorium formation is necessary for host penetration and colonization by *M. oryzae*. Previous studies have shown that hyphal-mediated infection of the host leaf by *M. oryzae* involves the pre-formation of an appressorium-like structure at the tip of the hyphae [12,45]. We reasoned out that the inability of ∆*Moget1*, ∆*Moget2* and ∆*Moget1/*∆*Moget2* mutants to cause infection might be due to their failure to form appressoria. To test this, we inoculated mycelial plugs from 5-day old cultures of the Guy11, the non-spore producing mutants ∆*Moget1*, ∆*Moget2* and ∆*Moget1*/∆*Moget2* on 10-day old barley (Golden Promise) leaves as well as on artificial hydrophobic cover slips, and incubated them in the dark for 24 hours. Unlike Guy11 and ∆*Moget1* mutant which produced appressoria on both natural and artificial hydrophobic surfaces, the ∆*Moget1/*∆*Moget2* double mutant failed to form any appressorium (Fig 6A). Few appressoria were formed on barley leaf surface, but not on artificial hydrophobic surface, when inoculated with ∆*Moget2* mutant mycelia plug (Fig 6A and 6C), suggesting that MoGet1/MoGet2 play an important synergistic role in appressorium formation. We also observed from the results that ∆*Moget1* and ∆*Moget2* mutants were able to penetrate the host leaf but failed to invade and cause disease symptoms after 48 to several hours of inoculation (Fig 6A).

To establish the reason for increased pathogenicity exhibited by ∆*Moget3* mutant, detached barley leaves were inoculated with conidia suspensions (1 × 10^4^ spores/mL) from Guy11, ∆*Moget3*, ∆*Moget4* and ∆*Mosgt2* strains. After 12, 16 and 24 h post-inoculation (hpi), we peeled the epidermal cells and observed for the development of invasive hyphae. All the strains breached the host cuticle after 12 h of infection, with ∆*Moget3* mutant developing more hyphal branches after 16 and 24 hpi (Fig 6B).

### MoGet1 and MoGet2 are required for hydrophobin synthesis

**Fig 7:**
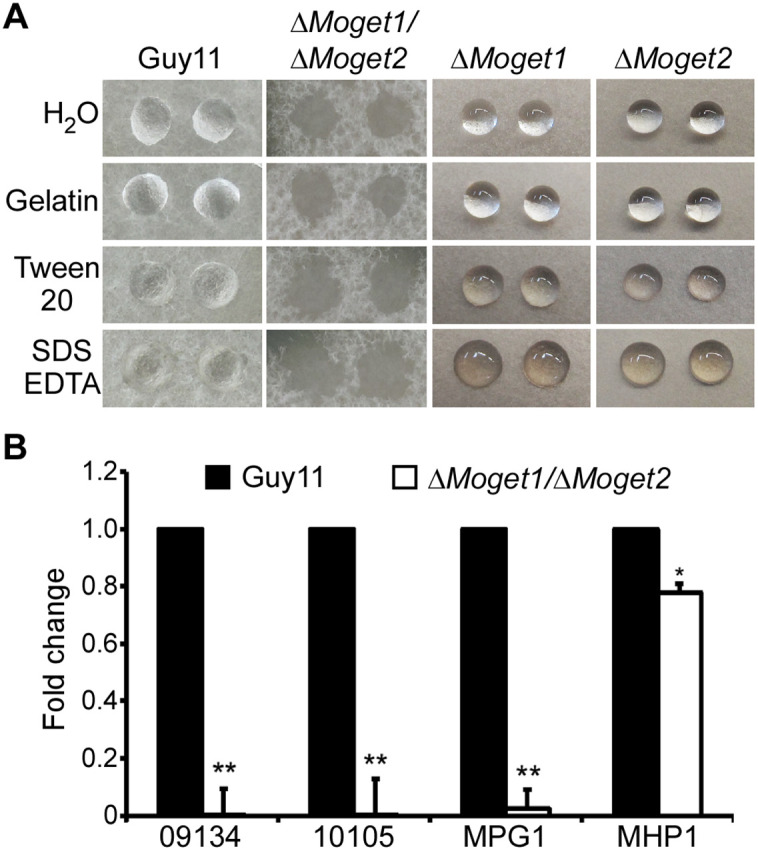
Detergent wettable phenotype of Guy11, ∆*Moget1*, ∆*Moget2* and ∆*Moget1/*∆*Moget2* in hydrophobicity solutions. **(A)** Hydrophobicity test on the hyphae of Guy11, ∆*Moget1/*∆*Moget2* and its single mutants in hydrophobicity test solutions. Surface hydrophobicity of the wild-type strain Guy11, ∆*Moget1*, ∆*Moget2* and ∆*Moget1*/∆*Moget2* mutant was assessed by placing 10 µl each of the test solution (ddH_2_O, 0.2% gelatine, 0.2% sodium dodecyl sulfate (SDS) + 50 mM EDTA, or 250 µg/ml Tween20) on the colony of the strains. The photographs were taken after 5 minutes. **(B)** Relative expression of hydrophobin-encoding genes, *MoMPG1*, *MoMHP1* and two *MoMHP1* homologues (MGG_09134 and MGG_10105) in the wild type strain Guy11 and ∆*Moget1*/∆*Moget2* double mutant. Error bars represent SD from the mean of three independent replicates and asterisks represent significant difference between Guy11 and ∆*Moget1*/∆*Moget2* mutant (** *p* < 0.01).

Since the ∆*Moget1/*∆*Moget2* mutant strain was unable to form appressoria on both artificial and natural hydrophobic surfaces, we speculated that failure to form appressorium could be attributed to its inability to sense and attach to their host’s hydrophobic surface due to lack of hydrophobin secretion [51–54]. To unravel this puzzle, we dropped 10 µl each of hydrophobicity testing solutions (ddH_2_O, 0.2% gelatin, 0.2% sodium dodecyl sulfate (SDS) + 50 mM EDTA) on the colony surfaces of Guy11, ∆*Moget1*, ∆*Moget2* and ∆*Moget1/*∆*Moget2* strains and incubated for 5 minutes. Results showed a retention of spherical shaped droplets solution on Guy11, ∆*Moget1* and ∆*Moget2* mycelia, but collapsed and spread on the ∆*Moget1/*∆*Moget2* mutant mycelia (Fig 7A)*.* This result suggests a compromise in hydrophobin secretion in the ∆*Moget1/*∆*Moget2* mutant. Furthermore, we checked the expression of genes involved in hydrophobin synthesis: *MoMPG1* (MGG_10315), *MoMHP1* (MGG_07047), MGG_10105 and MGG_09134 [54–56] in both Guy11 and the ∆*Moget1/*∆*Moget2* mutant. The expression of the four genes were significantly downregulated in ∆*Moget1*/∆*Moget*2 mutant compared to their expressions in wild type Guy11 strain (Fig 7B). Taken together, this result shows that MoGet1 and MoGet2 regulate hydrophobin biosynthesis in *M. oryzae*.

### MoGet1/MoGet2 complex regulates the autolysis and phosphorylation of Mps1

**Fig 8:**
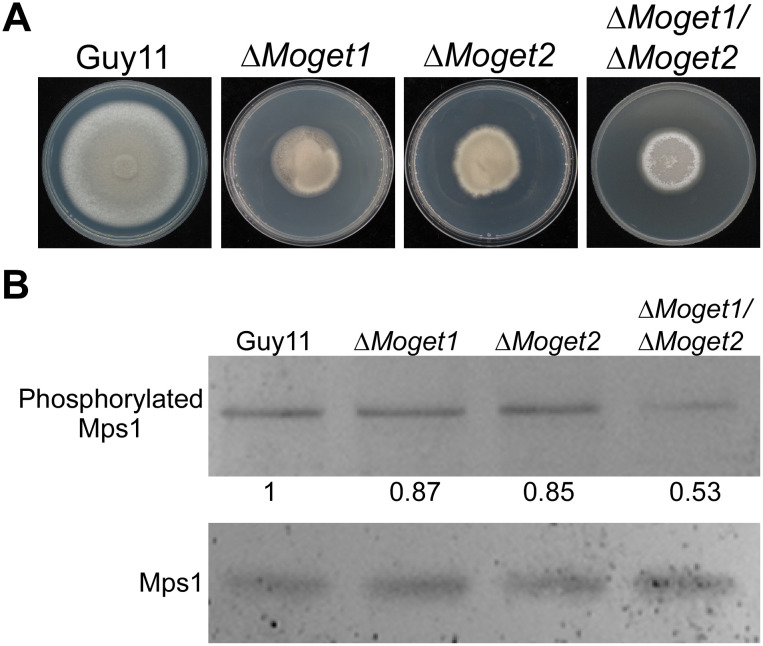
Autolysis and phosphorylation signal of ∆*Moget1/*∆*Moget2.* **(A)** Guy11 wild-type, ∆*Moget1*, ∆*Moget2* and ∆*Moget1/*∆*Moget2* cultured on CM II for 10 days. After 10 days, ∆*Moget1/*∆*Moget2* mycelial mass undergo heavy self-digestion at the distal region, unlike the other strains, suggesting that MoGet1 and MoGet2 act synergistically to regulate autolysis. **(B)** Western blot analysis of Mps1 MAPK phosphorylation in Guy11, ∆*Moget1,* ∆*Moget2* and ∆*Moget1/*∆*Moget2* double mutant. Phosphorylation of MoMps1 in Guy11 and ∆*Moget1/*∆*Moget2* was detected by p44/42 MAPK (Erk1/2) antibody. Total proteins were prepared from Guy11, ∆*Moget1, ∆Moget2* and ∆*Moget1/*∆*Moget2* mutant mycelia cultured in liquid CM medium. The phosphorylation level of MoMps1 in ∆*Moget1/*∆*Moget2* was significantly reduced by about 50% as depicted under the band. The phosphorylation level was quantified by scanning computer image analysis system using Tanon-5200 (Shanghai Co. Ltd, China).

Natural self-digestion of fungal mycelia normally occurs in aged filamentous fungal cultures. In our analysis of colony morphology of the strains, we observed autolysis in ∆*Moget1/*∆*Moget2* culture after 10 days of incubation (Fig 8A). This suggests that MoGet1 and MoGet2 jointly regulate ageing and preceding autolysis in *M. oryzae*.

Furthermore, Mps1 MAPK signaling cascade in *M. oryzae* is a popular pathway known to regulate cell wall biogenesis, fungal development and pathogenesis [57], and the activation of the pathway occurs through phosphorylation of MoMps1 [58]. To further investigate the roles of MoGet1/MoGet2 complex in *M. oryzae* development and determine whether the MoGet1 and MoGet2 are directly involved in Mps1 activation, we checked the phosphorylation level of Mps1 in the mutants. A western blot analysis revealed that, compared to the wild type, the phosphorylation level of Mps1 was significantly reduced by about 50% in the Δ*Moget1/*∆*Moget2* mutant (Fig 8B), indicating that MoGet1/MoGet2 plays an important synergistic role in regulating MoMps1 activation.

### MoGet1 and MoGet2 are localized to the ER while MoGet3, MoGet4 and MoSgt2 are cytosolic

**Fig 9:**
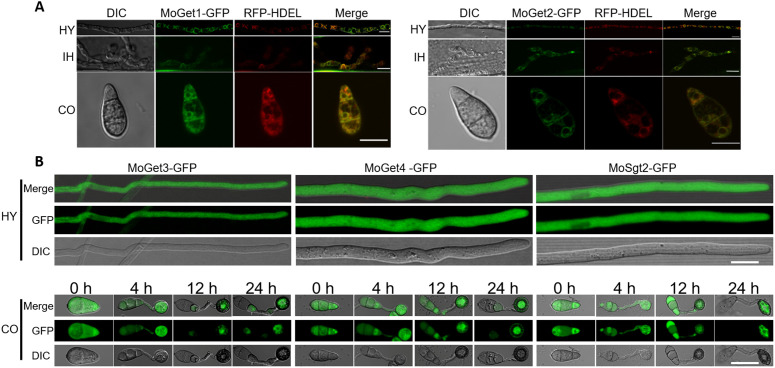
Subcellular localisation of MoGet1, MoGet2, MoGet3, MoGet4 and MoSgt2 at different stages of development. **(A)** Co-localization pattern of MoGet1 and MoGet2 in hyphae and conidia of *M. oryzae*. The MoGet1- and MoGet2-GFP do colocalize with the ER marker RFP-HDEL. **(B)** Localization of cytosolic MoGet3, MoGet4 and MoSgt2 in the hyphae, conidia and appressorium of *M. oryzae*. The live cell images were taken using Nikon Air Laser confocal microscopy. Scale bar = 10 µm.

To investigate the resident organelle of each of the five proteins under study, we fused *MoGET1*, *MoGET2*, *MoGET3*, *MoGET4* and *MoSGT2* along with their respective native promoters to a pYF11-GFP plasmid containing bleomycin resistant gene at the C-terminal. The constructs were transformed into the protoplasts of the respective mutants and selected on TB3 solid media supplemented with bleomycin sulfate. The fluorescence microscopy results showed that MoGet1 and MoGet2 were evenly distributed to a particular organelle while MoGet3, MoGet4 and MoSgt2 were clearly localized to the cytoplasm (Fig 9B). To ascertain the actual organelle to which MoGet1 and MoGet2 are localized, we transformed RFP-HDEL ER Marker into the strains and observed their hyphae and conidia under a laser scanning confocal microscope. We found that the GFP and RFP signals colocalized in both the hyphae and conidia (Fig 9A), suggesting the localization of MoGet1 and MoGet2 to the ER of *M. oryzae* and is supported by transmembrane domain that exist in the two proteins (Fig 1A and 1B).

### Y2H and co-immunoprecipitation assays suggest interaction among MoGet1, MoGet2 and MoGet3

**Fig 10:**
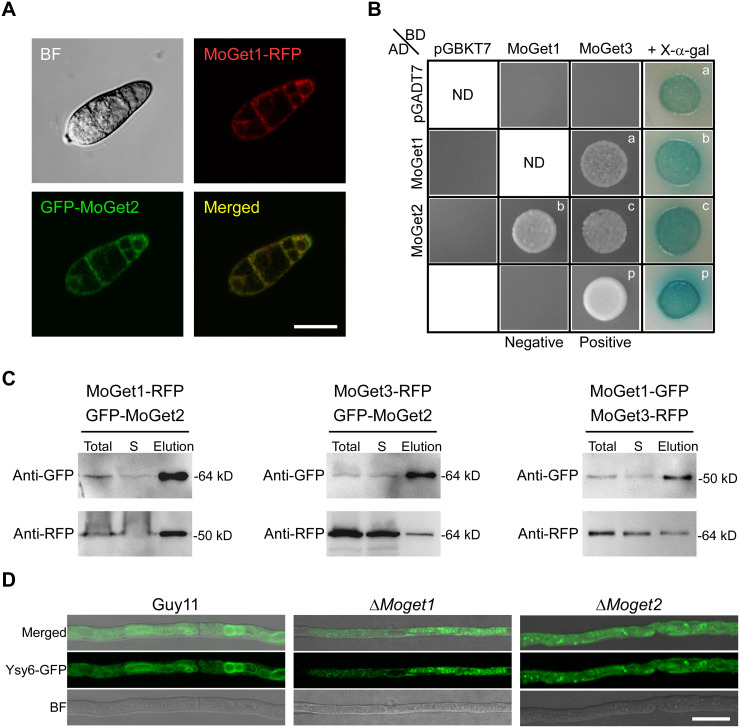
Interactions assay of MoGet1, MoGet2 and MoGet3, and localization of TA protein MoYsy6 in Guy11, ∆*Moget1* and ∆*Moget2.* (A) Confocal laser scanning showing co-localization of MoGet1-RFP and GFP-MoGet2 in spore of *M. oryzae*. Merge and individual fluorescence channels are shown. (B) Interaction between MoGet1 and MoGet2 and/or MoGet3 in rice blast fungus. (C) Western blot analysis of Co-Immunoprecipitated MoGet1, MoGet2 and MoGet3 tagged with GFP or RFP. (D) Determination of the fate of TA protein representative, MoYsy6, in Guy11, ∆*Moget1* and ∆*Moget2*.

The GFP and RFP fluorescence indicated that MoGet1 and MoGet2 were mainly localized to the ER (Figs 9A and 10A). Therefore, we hypothesized that the high expression and residence of MoGet1 and MoGet2 in the ER membrane could be due to existence of interaction between the two proteins. To test this hypothesis, we investigated the possible interaction between MoGet1 and MoGet2 or MoGet3 in *M. oryzae* via yeast two hybrid (Y2H) and Co-IP assays. The Y2H analysis showed that MoGet1 interacts with MoGet2 and MoGet3 (Fig 10B). Similarly, MoGet3 also showed positive interaction with MoGet2. Consistently, the results of the Co-IP assay confirmed that MoGet1 and MoGet2 interact with each other and with MoGet3 protein (Fig 10C). Therefore, we conclude that MoGet1 have direct relationship with MoGet2 and MoGet3, and likely forms a complex with MoGet2 on the ER membrane since MoGet1 and MoGet2 have transmembrane domain, unlike MoGet3 that lacks the domain (Fig 1).

### MoGet1 and MoGet2 regulate the localization of TA protein MoYsy6 in *M. oryzae*

In *S. cerevisiae*, Ysy6 and other ER-resident TA proteins were mislocalized in *get1/2*- and *get1*-null cells [16,20,59]. To reveal the fate of TA proteins in GET-deficient strains of *M. oryzae*, MoYsy6 was tagged with GFP, expressed in Guy11, ∆*Moget1* and ∆*Moget2* and observed using fluorescence microscopy. We found that while MoYsy6-GFP is localized to its resident organelle in Guy11, the loss of *MoGET1* or *MoGET2* leads to the mislocalization of MoYsy6-GFP, with the formation of a few puncta in the hyphal cell (Fig 10D).

## Discussion

In recent years, the structural, biochemical and functional characteristics of GET pathway components (such as Get1, Get2, Get3, Get4, Get5 and Sgt2) have been well studied in model yeast cell (*S. cerevisiae*) [16,60–62], mammals (*Rattus*) [28,63], *Plasmodium falciparum* [14] and *Arabidopsis thaliana* [64–66]. However, their functions in filamentous fungal pathogen *M. oryzae* is unknown. This study deployed functional genomic analysis and biochemical approach to elucidate the role of the five Get proteins (Get1, Get2, Get3, Get4, and Sgt2) in *M. oryzae*. Our domain architecture analysis shows that MoGet1 and MoGet2 have a transmembrane domain in contrast to MoGet3 and MoGet4, agreeing with the finding in yeast that ScGet3 and ScGet4 are cytosolic and do not require transmembrane domain [67–69]. However, unlike MoGet1, ScGet1 lack transmembrane domain (Fig 1A). MoSgt2 domain architecture, on the other hand, has TPR domain, which is conserved across all the strains analyzed. The TPR domain interacts with Ssa1 in *S. cerevisiae* to recruit TA proteins from the ribosome [70].

Upon deletion, our phenotypic functional analysis study showed that MoGet1 and MoGet2 are indispensable for vegetative growth and virulence of *M. oryzae*. The knockout mutants ∆*Moget1* and ∆*Moget2* were significantly retarded on CM, CM II, and all other solid media used for growth assay in this study, befitting the vital function of TA protein insertion [71]. The reduced growth exhibited by ∆*Moget1* on solid media is similar to the previous findings in *A. thaliana* where root hair elongation was impaired in *GET1*-deficient *Atget1* mutant strain [72]. This distorted vegetative growth could be attributed to TA mislocalization, toxicity of aggregated TA proteins in the cytosol, deficient mitophagy, or ER stress, as previously demonstrated in yeast cells and *A. thaliana* [59,73]. Although there is no clear report of a direct effect of Get1 and Get2 on filamentation in yeast cells, Zhang *et al* demonstrated that Gets-client protein Scs2 was essential for vegetative growth, asexual development and pathogenicity of *M. oryzae* [74]. ∆*Moget3,* on the other hand, had more expanded vegetative growth than Guy11 after 10 days of incubation. The increased vegetative growth displayed by ∆*Moget3* contrasts with the previous finding in *A. thaliana,* where the deletion of *AtGET3* resulted in the loss of root hairs and reduced growth [72], and in *Fusarium graminearum* whose ∆*Fgget3* deletion mutant is defective in vegetative growth and virulence [29], indicating a functional differentiation of Get3 protein in fungi.

Sporulation is critical in propagating and perpetuating the disease cycle of phytopathogenic fungi. In this study, our asexual reproduction analysis of each deletion mutant strain showed that ∆*Moget1*, ∆*Moget2* and their double deletion mutant are defective in conidiation. Unexpectedly, the loss of MoGet3 led to an increase in spore production by *M. oryzae*, suggesting that MoGet3 is a repressor of asexual reproduction in the rice blast fungus. This result is contrary to what was obtained in mammals, where the loss of TRC40/ASNA-1, Get3 ortholog in mammals led to embryonic lethality in mice [75] and impaired growth and insulin secretion in *Caenorhabditis elegans* [76].

In eukaryotes, osmotic stress leads to a response necessary for adaptation to hyperosmotic environments [77]. The adaptability of fungus and its propagules to diverse environmental changes (for instance, drought) and conditions is necessary for its survival and proliferation. In this study, our osmotic response test of GET complex factors established that mycelial growth of ∆*Moget2* was significantly inhibited in media supplemented with osmotic stressors 1 M KCl, 1 M NaCl and 1 M Sorbitol compared to Guy11 wild-type control (Fig 3 and Table 2). On sorbitol-supplemented CM II medium, ∆*Moget1* mutant had its inhibition rate significantly reduced compared to Guy11 wild-type (Fig 3 and Table 2), suggesting that MoGet1 and MoGet2 act antagonistically in the GET pathway to regulate sorbitol-mediated stress response in *M. oryzae.*

The fungal cell wall contains many of the pathogen-associated molecular patterns (PAMP) (for instance, chitin and β-glucan) recognized by host pattern recognition receptors (PRRs) during host-pathogen interactions [78,79]. During the interactions, one of the defence responses of the host plant is the release of ROS as an antimicrobial compound to restrict fungal invasion [80,81]. Although pathogenic fungi have evolved techniques to perceive and neutralize host-secreted enzymatic ROS [80], the overproduction of ROS (oxidative burst) inhibits biotrophic hyphal growth by causing localized cell death around the inoculation site [81]. In this study, our ROS stress response assay result established that ∆*Moget1/*∆*Moget2* was significantly inhibited on CM II media supplemented with 5 mM H_2_O_2_ ROS compared to its two single mutants, Guy11 and the other strains (Fig 3), suggesting that ∆*Moget1/*∆*Moget2* growth defect and failure to cause infection on the host could be, partly, attributed to its inability to sense and detoxify plant-produced ROS in the early stage of infection. It can be inferred that MoGet1 and MoGet2 are interdependent and involved in aiding the adaptation of *M. oryzae* to H_2_O_2_ stressor. The result demonstrates epistatic interactions in which the function of one gene is completely dependent on the presence of a second one [82,83]. This result is similar and expands on the previous finding in *S. cerevisiae,* where ∆*fus1/*∆*fus2*, ∆*fus1/*∆*spa1*, ∆*fus1/*∆*rvs161* and ∆*fus2/*∆*spa2* double mutants exhibited stronger cell fusion defects than their four single mutants [84].

Studies on genes involved in pathogenicity are essential for our overall knowledge of disease processes, and any such genes could be a target for disease control and management [85]. After disrupting the *GETs* genes, our pathogenicity assay on the host barley and rice leaves using spores or mycelial plugs established that both ∆*Moget1* and ∆*Moget2,* as well as their double knockout mutant ∆*Moget1/*∆*Moget2,* failed to cause the characteristic blast lesions on both wounded and unwounded host tissues (Fig 5A and 5B). More so, our penetration assay indicated that these mutants could develop penetration hyphae into the primary host cell but failed to branch into invasive hyphae (Fig 6A) after 1 – 3 weeks post-inoculation, suggesting that *MoGET1* and *MoGET2* are involved in invasion and pathogenicity. ∆*Moget3*, on the other hand, is more pathogenic than Guy11 wild-type. The penetration assay showed that ∆*Moget3* penetration peg could branch into invasive hyphae within 16 hpi, unlike the wild-type (Fig 6B). Increased virulence exhibited by ∆*Moget3* mimics previous findings where ∆*MoaclR* and ∆*Mobzip3* were more pathogenic than the wild type strain [4,86].

Hydrophobin coating mediates the attachment of fungi to host hydrophobic surfaces during host-pathogen interactions [55,87]. It can be suggested that ∆*Moget1/*∆*Moget2* failed in appressorium formation due to inability to produce hydrophobin (Fig 7B). The participation of the MoGet1 and MoGet2 in hydrophobin synthesis/hyphal hydrophobicity [87] may involve direct/indirect interaction between the proteins and the hydrophobic-related pathway. Further investigation is required to ascertain the crosstalk between hydrophobin genes and MoGet1/MoGet2 insertase in *M. oryzae* [59].

The MoGet1 and MoGet2 completely colocalized to the ER, while MoGet3, MoGet4, and MoSgt2 are localized to the cytoplasm. The co-localization of MoGet1 and MoGet2 to the ER is evident in the sensitivity of *MoGET1*- and *MoGET2*-deficient strains to ER stressor DTT and supports a synergistic role in the recruitment of Get3-TA protein complex to the ER lipid bilayer [44]. This result agrees with the previous finding, which established that *S. cerevisiae* ScGet1 and ScGet2 are ER-resident [59,88], while ScGet3, ScGet4, and ScSgt2 are cytosolic [27,89]. But, our domain architecture analysis showed that, unlike MoGet1, MoGet2 and ScGet2, ScGet1 lacks a transmembrane domain (Fig 1A and 1B). This observation suggests that while MoGet1 and MoGet2 reside and jointly recruit TA proteins into the ER lipid bilayer of *M. oryzae*, only ScGet2 of *S. cerevisiae* reside in ER, with ScGet1 possibly hanging in the cytosol to recruit TA protein substrates from ScGet3 and transfer it to ScGet2 in the *S. cerevisiae* ER membrane.

Most biological processes, including transcription regulation and signal transduction within and between cells, are governed by interactions between proteins [90], and the phenotype of an organism may be the combined output of multiple input signals. In this study, our Y2H assay and Co-IP analysis of MoGet1-RFP, MoGet1-GFP, GFP-MoGet2, and MoGet3-RFP demonstrated that MoGet1, MoGet2, and MoGet3 strongly interact with one another (Fig 10B and 10C), similar to earlier findings in *S. cerevisiae* and *A. thaliana* [16,44,72]. In *S. cerevisiae*, this interaction and the delivery of TA protein is ATP-dependent [16]. However, how these interactions occur in *M. oryzae* is yet unknown, so further investigation is needed.

Appropriate targeting of membrane proteins to correct subcellular destinations is essential for maintaining functional compartments within cells [59]. Our result of fluorescently tagged representative TA proteins, MoYsy6, in ∆*Moget1* and ∆*Moget2* reveals the mislocalization of the TA protein to different organelles (Fig 10D). This result confirms the demonstration where TA proteins are misdirected to the mitochondria [59] or aggregated in the cytosol in yeast cells [16,60]*.* This suggests the important biological role played by GET pathway components, MoGet1 and MoGet2, in the transfer of TA proteins to their destination organelles in the fungus.

In summary, we have demonstrated that GET complex components are conserved in *M. oryzae* and perform unique functions in vegetative growth, hydrophobicity, conidiation and pathogenicity. While MoGet4 and MoSgt2 have no significant function in *M. oryzae* pathogenicity, MoGet1 and MoGet2 are required for vegetative growth, conidiation and pathogenicity. We also demonstrated that MoGet2 is essential for cell wall integrity, osmotic and ER stress response and that MoGet1 and MoGet2 work synergistically to respond to H_2_O_2_ ROS stress. MoGet3, on the other hand, is a negative regulator of vegetative growth, conidiation and pathogenicity in *M. oryzae*. Moreover, we established that MoGet1 and MoGet2 colocalized to the ER and interacted with each other as well as with MoGet3. However, the live cell imaging of fluorescently labeled Get proteins indicates that MoGet3, MoGet4 and MoSgt2 are localized to the cytoplasm. Since MoGet3, MoGet4 and MoSgt2 are dispensable for vegetative growth, sporulation, appressorium formation, invasive growth and pathogenicity of *M. oryzae,* we suggest there is an alternative route for TA proteins, possibly from Ssa1 to ER membrane. This may be so because TA proteins must be transferred to their destination organelle to carry out their essential physiological functions of vesicular trafficking, protein translocation across organelles, programmed cell death, protein quality control and organelle dynamics and tethering [91]. Therefore, further research is needed to unravel the probable alternative pathway for TA proteins in *M. oryzae*.

## Supporting information

S1 Fig Confirmation of single insertion of hygromycin phosphotransferase gene (*HPH*) or bleomycin resistance gene (*Bleo*) in ORF region by Southern blot analysis. **A.** Probe1, Probe2, Probe3, Probe4 and Probe5 correspond to *MoGET1*, *MoGET2*, *MoGET3*, *MoGET4* and *MoSGT2* gene coding regions probe, respectively. Restriction enzymes used includes *Xho* I for probe1, probe 4, probe 5; *Hind* III for probe2 and *Eco*R V for probe 3. **B.** Genomic DNA extracted from wild-type control and the mutants were digested with restriction enzymes (*Eco*R V for ∆*Moget1* or ∆*Moget2*; *Eco*R I for ∆*Moget3*; *Xho* I for ∆*Moget4* and ∆*Mosgt2*) and hybridized with a probe specific for *HPH* fragment. **C.** Genomic DNA extracted from wild-type control and the double mutant were digested with restriction enzyme *Hind* III and hybridized with a probe specific for *Bleo* fragment.
(TIF)

S1 Table Primers used in this study. (DOCX)

S2 Table Gets in phytopathogenic fungi, *Neurospora crassa* and *Homo sapiens.* (DOCX)

S3 Table Cell wall and membrane integrity stress assay of MoGets component mutants. (DOCX)
