# Supplementary material for: Characterisation of guided entry of tail-anchored proteins in Magnaporthe oryzae
Source: PLoS Pathog. 2025 Jul 28;21(7):e1013011. doi: 10.1371/journal.ppat.1013011 (PMC12313070; doi:10.1371/journal.ppat.1013011)
Supplement: S1 Table — (DOCX) [file ppat.1013011.s002.docx]

**S1 Table. Primers used in this study.**

| Primer name | Primer Sequence (5' - 3') | Remark |
| --- | --- | --- |
| GET1 F1 | ACTATAGGGCGAATTGGGTACCTCGGTGATCATGCGGGTTG | Amplify *MoGET1* 5’ flank sequence |
| GET1 F2 | GCGCCGAATTCGATATCAAGCTTCTTTGGGGCTTCTGGGCTT | Amplify *MoGET1* 5’ flank sequence |
| GET1 F3 | TGCCGACCGGGCCGGCCGGATCCTTAGCCCAGAAGCTTGTCC | Amplify *MoGET1* 3’ flank sequence |
| GET1 F4 | GGTGGCGGCCGCTCTAGAGTAGTAGGGACTTGATACTA | Amplify *MoGET1* 3’ flank sequence |
| GET1 KO-F | GGCTAACTCATGCTGTTGGT | Amplify *MoGET1* probe sequence |
| GET1 KO-R | GAGCCACTCTGCGTAGTAC | Amplify *MoGET1* probe sequence |
| GET1 comF | ACTCACTATAGGGCGAATTGGGTACTCAAATTGGTTTCGACGCGATGGATGTGCTA | *MoGET1* complementation pYF11 |
| GET1 comR | CACCACCCCGGTGAACAGCTCCTCGCCCTTGCTCACTAGTTCCTTTTTGCCCTCC | *MoGET1* complementation pYF11 |
| GET1-RFP F | CTATAGGGCGAATTGGGTACTCAAATTGGTTTCGACGCGATGGATGTGCTA | Construction of Get1 RFP |
| GET1-RFP R | GAACTCCTTGATGACGTCCTCGGAGGAGGCTAGTTCCTTTTTGCCCTCC | Construction of Get1 RFP |
| GET2 F1 | ACTATAGGGCGAATTGGGTACCAACTGCAGAAGACCAGCAGT | Amplify *MoGET2* 5’ flank sequence |
| GET2 F2 | GCGCCGAATTCGATATCAAGCTTTTCTTTAGATTGTCGTGCACC | Amplify *MoGET2* 5’ flank sequence |
| GET2 F3 | TGCCGACCGGGCCGGCCGGATCCCTGAAAGAGATTATTGAGAGGG | Amplify *MoGET2* 3’ flank sequence |
| GET2 F4 | GGTGGCGGCCGCTCTAGAATTCACGCATTCCACCGCA | Amplify *MoGET2* 3’ flank sequence |
| GET2 KO-F | TAGGATCACTGGCCTTGGT | Amplify *MoGET2* probe sequence |
| GET2 KO-R | CCATCATCTGCTGCAGCAT | Amplify *MoGET2* probe sequence |
| GET2 comF | ACTCACTATAGGGCGAATTGGGTACTCAAATTGGTTCTACATCACCGAGGAGAACT | *MoGET2* complementation pYF11 |
| GET2 comR | CACCACCCCGGTGAACAGCTCCTCGCCCTTGCTCACAACACCAACAACCCCAATG | *MoGET2* complementation pYF11 |
| Get2 SQ-F1 | AACTGCAGAAGACCAGCAGT | Double knockout in ∆*Moget1* mutant |
| Get2 SQ-F2 | CAAAAATGCTCCTTCAGGTACCCTCGTTCTTTAGATTGTCGTGCACC | Double knockout in ∆*Moget1* mutant |
| Get2 SQ-F3 | GTTTACCTCTTCCAGATACAGCTCATCTCTGAAAGAGATTATTGAGAGGG | Double knockout in ∆*Moget1* mutant |
| Get2 SQ-F4 | ATTCACGCATTCCACCGCA | Double knockout in ∆*Moget1* mutant |
| GET3 F1 | ACTATAGGGCGAATTGGGTACCCACTGTTGGAGGCAAGAT | Amplify *MoGET3* 5’ flank sequence |
| GET3 F2 | GCGCCGAATTCGATATCAAGCTTGGTGGGGGAGCGTGTCCTTTA | Amplify *MoGET3* 5’ flank sequence |
| GET3 F3 | TGCCGACCGGGCCGGCCGGATCCAGAAATTGAATCTAAGGAGGG | Amplify *MoGET3* 3’ flank sequence |
| GET3 F4 | GGTGGCGGCCGCTCTAGATGGCAGCAACATGGGTGACT | Amplify *MoGET3* 3’ flank sequence |
| GET3 KO-F | GCGCATAGATCCCAGGAATA | Amplify *MoGET3* probe sequence |
| GET3 KO-R | GACGACGATGCAGTGAGTGT | Amplify *MoGET3* probe sequence |
| GET3 comF | ACTCACTATAGGGCGAATTGGGTACTCAAATTGGTTCAGCAACAGCGGAACATGT | *MoGET3* complementation pYF11 |
| GET3 comR | CACCACCCCGGTGAACAGCTCCTCGCCCTTGCTCACCTCGTCTGCCTCAGGGGGC | *MoGET3* complementation pYF11 |
| G3-RFP F | CTATAGGGCGAATTGGGTACTCAAATTGGTTCAGCAACAGCGGAACATGGT | Construction of Get3 RFP |
| G3-RFP R | GAACTCCTTGATGACGTCCTCGGAGGAGGCCTCGTCTGCCTCAGGGGGC | Construction of Get3 RFP |
| GET4 F1 | ACTATAGGGCGAATTGGGTACCAATCGCTTGACCTCCACGG | Amplify *MoGET4* 5’ flank sequence |
| GET4 F2 | GCGCCGAATTCGATATCAAGCTTGATGGATGGTTGAGGTTTGG | Amplify *MoGET4* 5’ flank sequence |
| GET4 F3 | TGCCGACCGGGCCGGCCGGATCCAGGGGTTTTATATTCGCAG | Amplify *MoGET4* 3’ flank sequence |
| GET4 F4 | GGTGGCGGCCGCTCTAGAGCATGCGACCAGTCAGTTA | Amplify *MoGET4* 3’ flank sequence |
| GET4 KO-F | CAAGACCGACAAGATCGACA | Amplify *MoGET4* probe sequence |
| GET4 KO-R | GACCAGCTAGTGATCGCACA | Amplify *MoGET4* probe sequence |
| GET4 comF | ACTCACTATAGGGCGAATTGGGTACTCAAATTGGTTTTGGCAGCTCTGCTGCGAC | *MoGET4* complementation pYF11 |
| GET4 comR | CACCACCCCGGTGAACAGCTCCTCGCCCTTGCTCACGTCTAATCCCTCTGCAGC | *MoGET4* complementation pYF11 |
| SGT2 F1 | ACTATAGGGCGAATTGGGTACCAAGACATGGGAGGGACGGT | Amplify *MoSGT2* 5’ flank sequence |
| SGT2 F2 | GCGCCGAATTCGATATCAAGCTTTGTTTGAATTTTCCGCCGTC | Amplify *MoSGT2* 5’ flank sequence |
| SGT2 F3 | TGCCGACCGGGCCGGCCGGATCCAGAATATAGTGGGTCTAAGCG | Amplify *MoSGT2* 3’ flank sequence |
| SGT2 F4 | GGTGGCGGCCGCTCTAGAGGCTGCCAGTCTACAATCTCAT | Amplify *MoSGT2* 3’ flank sequence |
| SGT2 KO-F | TACGAGAAGCTCAAGGGCA | Amplify *MoSGT2* probe sequence |
| SGT2 KO-R | AATGCTGGCCAAGCTGCTCA | Amplify *MoSGT2* probe sequence |
| SGT2 comF | ACTCACTATAGGGCGAATTGGGTACTCAAATTGGTTCAGATGTAGCAAACCACCTC | *MoSGT2* complementation pYF11 |
| SGT2 comR | CACCACCCCGGTGAACAGCTCCTCGCCCTTGCTCACCTGGCTACCCCGACCGGCAC | *MoSGT2* complementation pYF11 |
| MPG1 QRT_F | GAAGGTCGTCTCTTGCTGCA | Quantitative real-time PCR (qRT PCR) |
| MPG1 QRT_R | GGATGTTGACCAGACCAATC | Quantitative real-time PCR (qRT PCR) |
| MHP1 QRT_F | CACCATCATCGCCACCATC | Quantitative real-time PCR (qRT PCR) |
| MHP1 QRT_R | CAGCACTGAGCAGAGCCGTA | Quantitative real-time PCR (qRT PCR) |
| 09134 QRT_F | GCAGCGGAGCCTACAACAA | Quantitative real-time PCR (qRT PCR) |
| 09134 QRT_R | TCCAAGAACAGGGAGCAGACA | Quantitative real-time PCR (qRT PCR) |
| 10105 QRT_F | CGGCAGCGGAGACTATGA | Quantitative real-time PCR (qRT PCR) |
| 10105 QRT_R | CGCAAATGTCGGTGAAGC | Quantitative real-time PCR (qRT PCR) |
| BD-GET1 F | TCAGAGGAGGACCTGCATATGATGCCTTCACTACTGATTC | Yeast two hybrid |
| BD-GET1 R | TCGACGGATCCCCGGGAATTCTCATAGTTCCTTTTTGCCC | Yeast two hybrid |
| AD-GET1 F | GTACCAGATTACGCTCATATGATGCCTTCACTACTGATTC | Yeast two hybrid |
| AD-GET1 R | ATGCCCACCCGGGTGGAATTCTCATAGTTCCTTTTTGCCC | Yeast two hybrid |
| BD-GET2 F | TCAGAGGAGGACCTGCATATGATGACGGAATCAGCCGACGC | Yeast two hybrid |
| BD-GET2 R | TCGACGGATCCCCGGGAATTCTCAAACACCAACAACCCCA | Yeast two hybrid |
| AD-GET2 F | GTACCAGATTACGCTCATATGATGACGGAATCAGCCGACGC | Yeast two hybrid |
| AD-GET2 R | ATGCCCACCCGGGTGGAATTCTCAAACACCAACAACCCCA | Yeast two hybrid |
| BD-GET3 F | TCAGAGGAGGACCTGCATATGATGTCGACCGCCCTCATTGA | Yeast two hybrid |
| BD-GET3 R | TCGACGGATCCCCGGGAATTCCTACTCGTCTGCCTCAGGG | Yeast two hybrid |
| AD-GET3 F | GTACCAGATTACGCTCATATGATGTCGACCGCCCTCATTGA | Yeast two hybrid |
| AD-GET3 R | ATGCCCACCCGGGTGGAATTCCTACTCGTCTGCCTCAGGG | Yeast two hybrid |
