# Supplementary material for: Characterisation of guided entry of tail-anchored proteins in Magnaporthe oryzae
Source: PLoS Pathog. 2025 Jul 28;21(7):e1013011. doi: 10.1371/journal.ppat.1013011 (PMC12313070; doi:10.1371/journal.ppat.1013011)
Supplement: S2 Table — (DOCX) [file ppat.1013011.s003.docx]

**S2 Table. Gets in phytopathogenic fungi, *Neurospora crassa* and *Homo sapiens***

| **S/N** | **Organism** | **Protein name** | **Protein ID** |
| --- | --- | --- | --- |
| **1** | *Saccharomyces cerevisiae* | *S. cerevisiae* Get1 | NP_011495.1 |
|  |  | ScGet2 | NP_011006.2 |
|  |  | ScGet3 | NP_010183.1 |
|  |  | ScGet4 | NP_014807.3 |
|  |  | ScSgt2 | NP_014649.1 |
| **2** | *Magnaporthe oryzae* | *M. oryzae* Get1 | XP_003715385.1 |
|  |  | MoGet2 | XP_003717841.1 |
|  |  | MoGet3 | XP_003712205.1 |
|  |  | MoGet4 | XP_003715022.1 |
|  |  | MoSgt2 | XP_003710298.1 |
| **3** | *Botrytis cinerea* | *B. cinerea* Get1 | XP_024548203.1 |
|  |  | BcGet2 | EMR80383.1 |
|  |  | BcGet3 | XP_001552593.1 |
|  |  | BcGet4 | XP_024553867.1 |
|  |  | BcSgt2 | XP_001556769.1 |
| **4** | *Puccinia striiformis* | *P. striiformis* Get1 | KAI7967766.1 |
|  |  | PsGet2 | XP_047806390.1 |
|  |  | PsGet3 | KAI7934910.1 |
|  |  | PsGet4 | KNE97063.1 |
|  |  | PsSgt2 | XP_047807857.1 |
| **5** | *Fusarium graminearum* | *F. graminearum* Get1 | XP_011321211.1 |
|  |  | FgGet2 | QPC79602.1 |
|  |  | FgGet3 | XP_011318797.1 |
|  |  | FgGet4 | XP_011327068.1 |
|  |  | FgSgt2 | XP_011321549.1 |
| **6** | *Fusarium oxysporum* | *F. oxysporum* Get1 | KAG7407779.1 |
|  |  | FoGet2 | TXC07091.1 |
|  |  | FoGet3 | XP_018240895.1 |
|  |  | FoGet4 | XP_018234399.1 |
|  |  | FoSgt2 | KAH7214056.1 |
| **7** | *Blumeria graminis* | *B. graminis* Get1 | EPQ67104.1 |
|  |  | BgGet2 | CAD6504456.1 |
|  |  | BgGet3 | EPQ64508.1 |
|  |  | BgGet4 | EPQ61584.1 |
|  |  | BgSgt2 | EPQ62108.1 |
| **8** | *Zymoseptoria tritici* | *Z. tritici* Get1 | XP_003848188.1 |
|  |  | ZtGet2 | XP_003856636.1 |
|  |  | ZtGet3 | XP_003853017.1 |
|  |  | ZtGet4 | XP_003850056.1 |
|  |  | ZtSgt2 | XP_003857067.1 |
| **9** | *Colletotrichum fructicola* | *C. fructicola* Get1 | XP_036499662.1 |
|  |  | CfGet2 | XP_036499714.1 |
|  |  | CfGet3 | XP_036489687.1 |
|  |  | CfGet4 | XP_036498048.1 |
|  |  | CfSgt2 | XP_036493300.1 |
| **10** | *Ustilago maydis* | *U. maydis* Get1 | XP_011386800.1 |
|  |  | UmGet2 | XP_011391811.1 |
|  |  | UmGet3 | XP_011390272.1 |
|  |  | UmGet4 | XP_011387570.1 |
|  |  | UmSgt2 | XP_011392129.1 |
| **11** | *Neurospora crasa* | *N. crassa* Get1 | XP_957971.1 |
|  |  | NcGet2 | XP_963518.1 |
|  |  | NcGet3 | XP_960897.1 |
|  |  | NcGet4 | XP_961556.1 |
|  |  | NcSgt2 | XP_001728325.1 |
| **12** | *Homo sapiens* | *H. sapiens* Get1 | NP_004618.2 |
|  |  | HsGet2 | NP_001736.1 |
|  |  | HsGet3 | NP_004308.2 |
|  |  | HsGet4 | NP_057033.2 |
|  |  | HsSgt2 | NP_061945.1 |
