# Supplementary material for: Characterisation of guided entry of tail-anchored proteins in Magnaporthe oryzae
Source: PLoS Pathog. 2025 Jul 28;21(7):e1013011. doi: 10.1371/journal.ppat.1013011 (PMC12313070; doi:10.1371/journal.ppat.1013011)
Supplement: S3 Table — (DOCX) [file ppat.1013011.s004.docx]

**S3 Table. Cell wall and membrane integrity stress assay of MoGets component mutants**

|  | **Inhibition rate (%)** | | | |
| --- | --- | --- | --- | --- |
| **Strain** | CR | SDS | DTT | CFW |
| Guy11 | 22.03±3.64^c^ | 68.94±13.53^a^ | 3.17±1.39^d^ | 16.90±8.68^d^ |
| ∆*Moget1/*∆*Moget2* | 26.89±13.10^b^ | 44.38±22.18^c^ | 1.32±4.26^e^ | 26.26±17.10^c^ |
| ∆*Moget1* | 24.16±16.46^c^ | 60.76±4.88^b^ | 28.19±7.47^b^ | 23.99±24.75^c^ |
| ∆*Moget1/MoGET1* | 23.21±11.18^c^ | 64.88±11.94^b^ | 3.47±0.12^d^ | 12.81±2.09^d^ |
| ∆*Moget2* | 29.07±6.41^b^ | 62.26±12.45^b^ | 37.02±20.68^a^ | 42.16±7.45^a^ |
| ∆*Moget2/MoGET2* | 28.05±3.34^b^ | 64.57±8.93^b^ | 9.43±8.63^c^ | 27.21±0.21^c^ |
| ∆*Moget3* | 11.49±8.04^e^ | 68.55±2.96^a^ | 4.58±3.56^d^ | 15.36±0.93^d^ |
| ∆*Moget3/MoGET3* | 23.39±2.66^c^ | 60.57±12.52^b^ | 3.51±0.40^d^ | 10.05±1.10^d^ |
| ∆*Moget4* | 17.09±5.87^d^ | 62.13±12.69^b^ | 4.42±7.88^d^ | 11.90±7.26^d^ |
| ∆*Moget4/MoGET4* | 24.69±0.44^c^ | 73.09±4.93^a^ | 3.26±0.81^d^ | 24.95±6.15^c^ |
| ∆*Mosgt2* | 10.73±5.76^e^ | 65.15±9.32^b^ | 13.29±10.70^c^ | 21.95±8.70^c^ |
| ∆*Mosgt2/MoSGT2* | 35.91±1.37^a^ | 62.38±4.59^b^ | 3.23±1.66^d^ | 33.82±6.25^b^ |
| Growth inhibition rate of Guy11, ∆*Moget1/*∆*Moget2,* ∆*Moget1*, ∆*Moget2*, ∆*Moget3*, ∆*Moget4* and ∆*Mosgt2* and their complemented strains on CR, SDS, DTT and CFW | | | | |
| Mean and standard deviation were determined using contingency table analysis with row means in Microsoft Excel spreadsheets and GraphPad Prism 5. Similar values were obtained from three independent experimental repeats with 3 technical replicates for each repetition. | | | | |
| The inhibition rate of each treatment was compared with the growth rate of the untreated control. Inhibition rate = (the colony diameter of untreated strain—the colony diameter of the treated strain)/the colony diameter of untreated strain) * 100. The superscript a, b, c, d and e indicate significant changes compare to Guy11. Same letters in a column shows no significant difference. | | | | |
